# Paper-Based Survivorship Care Plans May be Less Helpful for Cancer Patients Who Search for Disease-Related Information on the Internet: Results of the Registrationsystem Oncological Gynecology (ROGY) Care Randomized Trial

**DOI:** 10.2196/jmir.4914

**Published:** 2016-07-08

**Authors:** Kim AH Nicolaije, Nicole PM Ezendam, Johanna MA Pijnenborg, Dorry Boll, Maria Caroline Vos, Roy FPM Kruitwagen, Lonneke V van de Poll-Franse

**Affiliations:** ^1^ Center of Research on Psychology in Somatic diseases (CoRPS) Department of Medical and Clinical Psychology Tilburg University Tilburg Netherlands; ^2^ Comprehensive Cancer Center The Netherlands Eindhoven Netherlands; ^3^ Gynecologic Cancer Center South Department of Gynecology Elisabeth-TweeSteden Hospital Tilburg and Waalwijk Netherlands; ^4^ Department of Gynecology and GROW School for Oncology and Developmental Biology Maastricht University Medical Center Maastricht Netherlands

**Keywords:** Survivorship Care Plan, Internet use, pragmatic cluster randomized trial, endometrial neoplasms, patient-reported outcomes, information provision

## Abstract

**Background:**

The Institute of Medicine recommends Survivorship Care Plans (SCPs) for all cancer survivors. However, it is unclear whether certain patient groups may or may not benefit from SCPs.

**Objective:**

The aim was to assess whether the effects of an automatically generated paper SCP on patients’ satisfaction with information provision and care, illness perceptions, and health care utilization were moderated by disease-related Internet use.

**Methods:**

Twelve hospitals were randomized to either SCP care or usual care in the pragmatic cluster randomized Registrationsystem Oncological GYnecology (ROGY) Care trial. Newly diagnosed endometrial cancer patients completed questionnaires after diagnosis (N=221; response: 74.7%, 221/296), 6 months (n=158), and 12 months (n=147), including patients’ satisfaction with information provision and care, illness perceptions, health care utilization (how many times patients visited a medical specialist or primary care physician about their cancer in the past 6 months), and disease-related Internet use (whether patients used the Internet to look for information about cancer).

**Results:**

In total, 80 of 221 (36.2%) patients used the Internet to obtain disease-related information. Disease-related Internet use moderated the SCP care effect on the amount of information received about the disease (*P*=.03) and medical tests (*P*=.01), helpfulness of the information (*P*=.01), and how well patients understood their illness (*P*=.04). All stratified analyses were not statistically significant. However, it appeared that patients who did not seek disease-related information on the Internet in the SCP care arm reported receiving more information about their disease (mean 63.9, SD 20.1 vs mean 58.3, SD 23.7) and medical tests (mean 70.6, SD 23.5 vs mean 64.7, SD 24.9), finding the information more helpful (76.7, SD 22.9 vs mean 67.8, SD 27.2; scale 0-100), and understanding their illness better (mean 6.6, SD 3.0 vs mean 6.1, SD 3.2; scale 1-10) than patients in the usual care arm did. In addition, although all stratified analyses were not significant, patients who did seek disease-related information on the Internet in the SCP care arm appeared to receive less information about their disease (mean 65.7, SD 23.4 vs mean 67.1, SD 20.7) and medical tests (mean 72.4, SD 23.5 vs mean 75.3, SD 21.6), did not find the information more helpful (mean 78.6, SD 21.2 vs mean 76.0, SD 22.0), and reported less understanding of their illness (mean 6.3, SD 2.8 vs mean 7.1, SD 2.7) than patients in the usual care arm did.

**Conclusions:**

Paper SCPs appear to improve the amount of information received about the disease and medical tests, the helpfulness of the information, and understanding of the illness for patients who do not search for disease-related information on the Internet. In contrast, paper SCPs do not seem beneficial for patients who do seek disease-related information on the Internet.

**Trial Registration:**

ClinicalTrials.gov NCT01185626; https://clinicaltrials.gov/ct2/show/NCT01185626 (Archived by WebCite at http://www.webcitation.org/6fpaMXsDn)

## Introduction

Information provision has been demonstrated to play an essential role in the support for cancer survivors [1,2]. To improve patient information provision, the Institute of Medicine (IOM) recommends the use of Survivorship Care Plans (SCPs), described as personal treatment summaries and follow-up care plans, for all cancer survivors [3]. However, there is still an ongoing debate about the benefits of SCPs [4-12].

Recent results of the pragmatic cluster randomized Registrationsystem Oncological GYnecology (ROGY) Care trial [8], in which cancer patients were provided with a paper-based SCP, showed that SCPs increased the amount of information received. However, the trial showed no evidence of SCPs benefitting satisfaction with information and care. Furthermore, SCPs increased patients’ concerns, emotional impact, experienced symptoms, and the amount of cancer-related contact with the primary care physician. Moreover, it remains unclear whether patient characteristics influence the effects of SCPs and whether certain groups of patients may or may not benefit from SCPs [8].

The SCPs are usually provided by patients’ health care providers, who are patients’ main source of information about their cancer [1,13]. However, the Internet is also increasingly used as a source of information. Several studies have shown that a significant proportion of cancer survivors, ranging from 30% to 60%, are using the Internet to seek information about their cancer [14-19]. Especially those cancer survivors who are younger [15,17,19], higher educated [15,17,19], male [15], and have a partner [19] use the Internet.

Using the Internet to obtain disease-related information has been associated with considerable benefits for cancer survivors [20]. For instance, it has been found that cancer survivors who use the Internet to access disease-related information feel better informed [15], report receiving more information about their disease and medical tests [21], find the received information more helpful [21], communicate more effectively with their health care providers [22], and are more actively involved in decision making [23]. Therefore, it is possible that receiving an SCP has a different impact on patients who search for information about their cancer on the Internet compared to patients who do not search for information about their cancer on the Internet.

The ROGY Care trial evaluates the impact of an automatically generated SCP on outcomes reported by gynecological cancer patients and health care providers. The trial protocol [24], the primary patient-reported outcomes up to 12 months after diagnosis [8], and the evaluation of the oncology providers [28] and primary care physicians [46] have been previously described. The aim of this analysis of the ROGY Care trial was to assess whether the effects of an automatically generated paper SCP on patients’ satisfaction with information provision and care, illness perceptions, and health care utilization were moderated by (ie, different for) disease-related Internet use. It was hypothesized that paper SCPs may be a helpful tool to reach out to patient groups who do not search for information about their cancer on the Internet, whereas SCPs may be of limited value for patients who already benefit from accessing information about their cancer on the Internet.

## Methods

### Design

In the pragmatic cluster randomized controlled ROGY Care trial, 12 hospitals in the Netherlands were randomized to either SCP care or usual care. Patients were included immediately after initial surgery and followed for 24 months. The trial was centrally approved by the Medical Research Ethics Committee of the St Elisabeth Hospital in Tilburg, as well as by the Medical Research Ethics Committees of each participating center [24], and has been registered on ClinicalTrials.gov (NCT01185626). This study describes the results of subgroup analyses of the primary patient-reported outcomes up to 12 months after diagnosis.

### Participants and Recruitment

Participants were women newly diagnosed with endometrial cancer. Exclusion criteria (ie, undergoing palliative care or unable to complete a Dutch questionnaire) [24] were minimal to maximize generalizability [25]. Between April 2011 and October 2012, all eligible patients were invited to participate after initial diagnosis by their own gynecologist by sending a letter, questionnaire, and informed consent form [8,24]. After the first contact through the gynecologist and obtaining informed consent, follow-up questionnaires were sent directly to the home address of the patient at 6 and 12 months after diagnosis *.*

### Randomization and Blinding

Randomization at the hospital level was chosen to avoid potential contamination of usual care with increased information provision of SCP care and was performed with a table of random numbers by a researcher not involved in the study and blind to the identity of the hospitals. As is common in cluster randomized trials [27], patients were unaware of the assignment to trial arms. Health care providers could not be blinded to trial arm assignment.

### Survivorship Care Plan Versus Usual Care

In the usual care arm, the oncology providers (ie, gynecologists, gynecologic oncologists, oncology nurses) were instructed to continue providing patient information in the way they were used to: they gave standard care according to the Dutch follow-up guidelines, which recommend verbal and written information about the period after treatment and follow-up, signs of recurrence, and hospital contact details. None of the oncology providers in the usual care arm provided SCPs [28].

In the SCP care arm, the oncology providers were instructed to provide an SCP to patients after surgery (ie, during the consultation in which the final histological diagnosis was discussed); to provide an updated SCP during follow-up visits if there were changes in the cancer, treatment, or specialists; and to send a copy of the SCP to the patient’s primary care physician. Because of the pragmatic approach of the trial, the delivery of the intervention was allowed to vary between hospitals and oncology providers, fitting their own clinical practice [24].

### Survivorship Care Plan

The Web-based ROGY has been used by all participating oncology providers in both arms since 2006. For each patient, a detailed registration is made in a uniform way, including tumor stage and grade, treatment, comorbidity, complications, follow-up, and information about the involved specialists (eg, gynecologist/gynecologic oncologist, medical oncologist, radiation oncologist). For this trial, an application was built in ROGY enabling automatic generation of an SCP combining patient and disease data by simply pressing a button. The ROGY system was used by all participating oncology providers in both arms, but the SCP button was only visible for oncology providers in the SCP care arm. Any changes related to the cancer, treatment, or specialists were registered in ROGY and automatically updated in the SCP during follow-up.

For the development of the SCP, the Dutch SCP template (based on the IOM format) [3], was adjusted to the local situation [29] by a subgroup of gynecologists/gynecologic oncologists, oncology nurses, a radiation oncologist, a medical oncologist, a primary care physician, and patients [24]. The SCP was pilot-tested on patients with a low/intermediate educational level to ensure that the SCP was understandable.

The SCP consisted of a tailored treatment summary, including information on diagnostic tests, type of cancer, stage, grade, treatment, and contact details of the hospital and specialists. In addition, the SCP contained a tailored follow-up care plan, including detailed information on possible short-term and long-term effects, effects on social and sexual life, possible signs of recurrence and secondary tumors, and information on rehabilitation, psychosocial support, and supportive care services [24].

### Measures

All questionnaires were assessed after initial diagnosis and after 6 and 12 months.

#### Moderator Variable

Disease-related Internet use was assessed by asking whether patients had used the Internet to look for information about cancer, which could be answered by either yes or no.

#### Dependent Variables

Satisfaction with information provision was assessed with the European Organisation for Research and Treatment of Cancer (EORTC) Quality of Life Group information (QLQ-INFO25) questionnaire [30]. This questionnaire includes four information provision subscales: perceived receipt of information about the disease (four items regarding diagnosis, spread of disease, cause(s) of disease, and whether the disease is under control), medical tests (three items regarding purpose, procedures, and results of tests), treatment (six items regarding medical treatment, benefits, side effects, effects on disease symptoms, social life, and sexual activity) and other care services (four items regarding additional help, rehabilitation options, managing illness at home, psychological support). The question format was as follows: “During your current disease or treatment, how much information have you received on...?” In addition, four single-items were included (information about different places of care, things you can do to help yourself get well, satisfaction with the information, and helpfulness of the information). The answer categories were “not at all,” “a little,” “quite a bit,” and “very much.” The scales were converted to 0-100 linear scales, with higher scores indicating better-perceived information provision. Internal consistency for all scales (Cronbach alphas=.70-.87) and test-retest reliability (intraclass correlations=.71-.91) were good [30].

Satisfaction with care was assessed with two multi-item and two single-item scales of the EORTC cancer in-patient satisfaction with care measure (IN-PATSAT32) [31]. This questionnaire was designed to assess cancer patients’ perception of the quality of medical care, nursing care, and care organization and services received in the hospital. The multi-item scales included doctors’ and nurses’ interpersonal skills. The single-item scales included exchange of information between caregivers and general satisfaction with care. The question format was as follows: “How would you rate...?” The answer categories were “poor,” “fair,” “good,” “very good,” and “excellent.” The scales were converted to 0-100 linear scales, with higher scores indicating better-perceived quality of care. Internal consistency (Cronbach alphas=.67-.96) and test-retest reliability (intraclass correlations=.66-.85) were good [31].

Illness perception was assessed with the Brief Illness Perception Questionnaire (B-IPQ) [32], consisting of eight single-item scales, measuring cognitive representations (consequences, timeline, personal control, treatment control, identity), emotional representations (concern, emotion), and illness comprehensibility rated on a 0-10 linear scale, with higher scores indicating more endorsement of that item. Test-retest reliability (Pearson correlations=.42-.75) was good [32].

Health care utilization was assessed by asking how many times patients visited a medical specialist or primary care physician in relation to cancer in the past 6 months. These questions were asked in a similar way as is done by Statistics Netherlands.

#### Control Variables

Sociodemographic and clinical information were obtained from ROGY (ie, date of birth, date of diagnosis, disease stage, primary treatment) and the questionnaire (ie, marital status, educational level as an indicator for socioeconomic status [SES], employment status). Comorbidity was assessed by the adapted Self-administered Comorbidity Questionnaire (SCQ) [33].

### Statistical Analyses

Statistical analyses were conducted using SPSS version 19.0 (IBM Corp, Armonk, NY, USA). Tests were two-sided and considered significant if *P*<.05. Both intention-to-treat and per protocol analyses were conducted. Intention-to-treat analyses compared all respondents in the SCP care arm to all respondents in the usual care arm. Per protocol analyses compared respondents in the SCP care arm who indicated receiving an SCP in the first questionnaire to all respondents in the usual care arm. Because intention-to-treat and per protocol analyses revealed similar results, only the results of the intention-to-treat analyses are reported in this study.

Means with standard deviations were used to describe continuous variables and frequencies with percentages to describe categorical variables. Differences in sociodemographic and clinical characteristics between respondents and nonrespondents, between the SCP care arm and the usual care arm, and between patients who did or did not use the Internet to obtain information about their disease were compared using *t* tests for continuous variables and chi-square tests for categorical variables.

Moderation of disease-related Internet use on the dependent variables (ie, 22 scales in total: eight on information provision, four on satisfaction with care, eight on illness perceptions, and two on health care utilization) was tested by assessing the significance of the interaction term “trial arm × disease-related Internet use” in the overall linear multilevel regression model. Multilevel analysis corrects for missing data (assumed missing at random) by using information from the observed outcomes to provide information about the unobserved outcomes [34,35]

The model included two random intercepts (ie, hospital- and patient-level) to account for both clustering at hospital-level and intrapatient dependency of repeated measures [36], the independent variables intervention arm (ie, SCP care vs usual care) and time, the covariates age, time since diagnosis, marital status, employment, educational level, comorbidity, disease stage, and treatment, and the dependent variables information provision and care, illness perceptions, and health care utilization. For the models that did not converge, hospital was included as covariate instead of as random intercept [37].

When an interaction term was significant, this was an indication that the effect of providing an SCP was different for patients who did or did not use the Internet to search for disease-related information and that stratified analyses were warranted to further explore the direction of the moderation effects. For significant interaction terms, the intervention effects were re-examined in subgroups by performing the overall linear multilevel regression analyses stratified by the levels of the moderator variable (ie, disease-related Internet use). Unstandardized betas were presented with 95% confidence intervals.

The trial was originally powered to detect a clinically meaningful difference on the overall primary outcomes of the intervention, targeting 75 patients per arm [8,24]. The trial was not powered to detect differences in moderation analyses or stratified analyses. In this study, moderation analyses and stratified analyses were performed despite this lack of power because we merely wanted to explore the potential moderating role of Internet use. These analyses can be justified because they are exploratory and because the exploration was a priori restricted to a selected moderator with a specific rationale [38].

## Results

### Patient Characteristics

**Figure 1:**
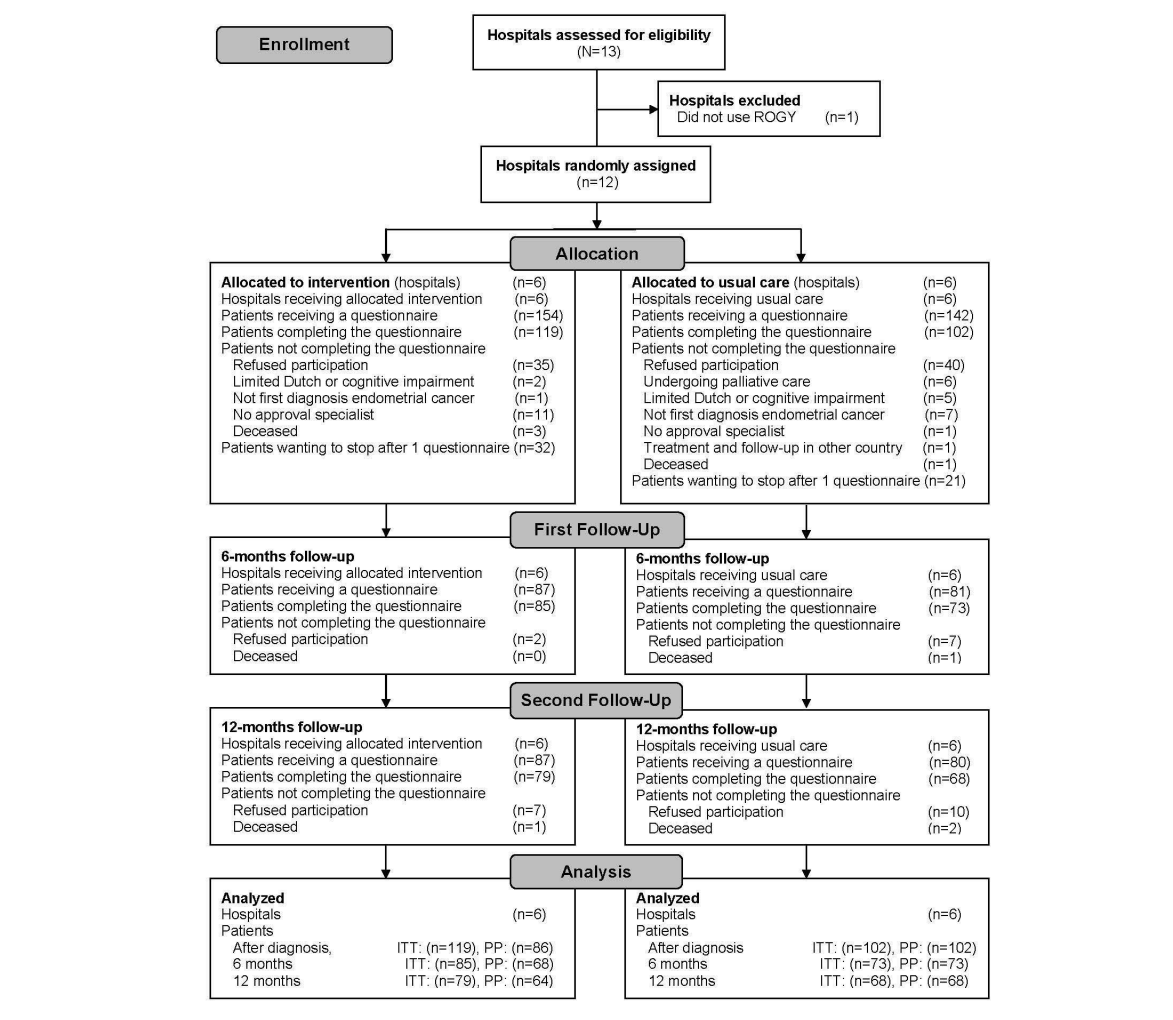
CONSORT flow diagram of the progress of the hospitals and endometrial cancer patients through the phases of the ROGY Care trial. ITT: intention-to-treat analyses (comparing all respondents in the SCP care arm to all respondents in the usual care arm); PP: per protocol analyses (comparing the respondents in the SCP care arm who indicated that they received an SCP in the first questionnaire to all respondents in the usual care arm).

Of the 296 eligible patients, 221 (74.7%) patients completed the first questionnaire. After 6 months, 158 patients completed the questionnaire; after 12 months, 147 patients completed the questionnaire (Figure 1) [8].

At baseline, participants were younger (mean 67.4, SD 8.9 years) than nonparticipants (mean 70.2, SD 9.5 years, *P*=.02), and more often had an International Federation of Gynecology and Obstetrics (FIGO) staging level of stage I (85.5%, 189/221 vs 69%, 52/75; *P*=.003; Table 1) [8]. In total, 80 of 221 (36.2%) patients indicated that they used the Internet to obtain information about their disease. This did not differ between the SCP care arm and the usual care arm (Table 2).

**Table 1:** CONSORT table of baseline sociodemographic and clinical characteristics of participants according to trial arm and of nonparticipants.

Patient characteristics		SCP care (n=119)	Usual care (n=102)	*P* ^a^	Total participants (N=221)	Nonparticipants (n=75)	*P* ^a^
Age at diagnosis, mean (SD)		67.1 (9.1)	67.7 (8.8)	.65	67.4 (8.9)	70.2 (9.5)	.02
**FIGO stage, n (%)**							
	I	102 (85.7)	87 (85.3)	.75	189 (85.5)	52 (69)	.003
	II	5 (4.2)	2 (2.0)		7 (3.2)	10 (13)	
	II	8 (6.7)	7 (6.8)		15 (6.8)	11 (15)	
	IV	3 (2.9)	4 (3.9)		7 (3.2)	1 (1)	
**Treatment, n (%)**							
	Surgery	117 (98.3)	97 (95)	.46	214 (96.8)	72 (96)	.45
	Radiotherapy	44 (37.0)	37 (36.3)	.99	81 (36.7)	34 (45)	.19
	Chemotherapy	6 (5.0)	12 (11.8)	.06	18 (8.1)	7 (9)	.76
**Hospital, n (%)**							
	1	22 (18.5)			22 (10.0)	4 (5)	.61
	2	12 (10.1)			12 (5.4)	7 (9)	
	3	28 (23.5)			28 (12.7)	9 (12)	
	4	28 (23.5)			28 (12.7)	9 (12)	
	5	11 (9.2)			11 (5.0)	1 (1)	
	6	18 (15.1)			18 (8.1)	5 (7)	
	7		25 (24.5)		25 (11.3)	13 (17)	
	8		21 (20.5)		21 (9.5)	6 (8)	
	9		26 (25.5)		26 (11.8)	7 (9)	
	10		12 (11.8)		12 (5.4)	4 (5)	
	11		3 (2.9)		3 (1.4)	3 (4)	
	12		15 (14.7)		15 (6.8)	7 (9)	

^a^*P* values report comparisons between the intervention arm and the usual care arm, and between the trial participants and nonparticipants according to *t* tests and chi-square tests.

**Table 2:** Sociodemographic and clinical characteristics at the first questionnaire according to trial arm.

Patient characteristics		SCP care (n=119)		Usual care (n=102)		*P* ^a^		Total (N=221)	
Age at time of survey, mean (SD)		67.4 (9.1)		67.8 (8.9)		.71		67.6 (9.0)	
Months since diagnosis, mean (SD)		2.6 (1.7)		1.8 (1.2)		<.001		2.1 (1.5)	
**Months since diagnosis, n (%)**									
	<1	12 (10.1)		24 (23.5)				36 (16.3)
	1-2	40 (33.6)		46 (45.0)				86 (38.9)
	2-3	33 (27.7)		20 (19.6)				53 (24.0)
	>3	34 (28.6)		12 (11.8)				46 (20.8)
**Comorbidity, n (%)**						.41			
	None	19 (16.0)		18 (17.6)				37 (16.7)
	1	32 (26.9)		20 (19.6)				52 (23.5)
	≥2	64 (53.8)		62 (60.8)				126 (57.0)
**Marital status,** ^b^ **n (%)**						.74			
	Partner	85 (71.4)		76 (74.5)				161 (72.9)
	No partner	31 (26.1)		25 (24.5)				56 (25.3)
**Educational level,^c^n (%)**						.09			
	High	17 (14.3)		7 (6.9)				24 (10.9)
	Intermediate	71 (59.7)		72 (70.6)				143 (64.7)
	Low	30 (25.2)		19 (18.6)				49 (22.2)
**Employed, n (%)**						.40			
	Yes	22 (18.5)		15 (14.7)				37 (16.7)
	No	85 (71.4)		79 (77.5)				164 (74.2)
**Disease-related Internet use, n (%)**						.57			
	Yes	41 (34.5)		39 (38.2)				80 (36.2)
	No	76 (63.9)		60 (58.8)				136 (61.5)

^a^*P* values report comparisons between the intervention arm and the usual care arm, according to *t* tests and chi-square tests.

^b^Marital status: partner=married/living together, no partner=divorced/widowed/never married.

^c^Educational level: low=no/primary school, intermediate=lower general secondary education/vocational training, high=preuniversity education/ high vocational training/university.

Patients who used the Internet to obtain disease-related information were younger (mean 62.8, SD 7.5 years) than patients who did not use the Internet to obtain disease-related information (mean 70.3, SD 8.7 years; Table 3). In addition, patients who used the Internet to obtain disease-related information more often had a partner (83%, 66/80 vs 67.6%, 92/136), more often had a high educational level (20%, 16/80 vs 5.1%, 7/136), and were employed more often (28%, 21/80 vs 11.8%, 16/136) than patients who did not use the Internet to obtain disease-related information.

**Table 3:** Patient characteristics at the first questionnaire according to disease-related Internet use.

Patient characteristics			Disease-related Internet use (n=80)		No disease-related Internet use (n=136)		*P* ^a^		Total (N=216)
Age at time of survey, mean (SD)			62.8 (7.5)		70.3 (8.7)		<.001		67.5 (9.0)
Months since diagnosis, mean (SD)			2.0 (1.3)		2.3 (1.6)		.10		2.2 (1.5)
**Months since diagnosis, n (%)**									
	<1		19 (24)		16 (11.8)				35 (16.2)	
	1-2		31 (39)		53 (39.0)				84 (38.9)	
	2-3		12 (15)		41 (30.1)				53 (24.5)	
	>3		18 (23)		26 (19.1)				44 (20.4)	
**FIGO stage, n (%)**							.37		
	I		70 (88)		115 (84.6)				185 (85.6)	
	II		4 (5)		3 (2.2)				7 (3.2)	
	II		3 (4)		12 (8.8)				15 (6.9)	
	IV		2 (3)		4 (2.9)				6 (2.8)	
**Treatment, n (%)**									
	Surgery		77 (96)		132 (97.1)		.90		209 (96.8)	
	Radiotherapy		26 (33)		54 (39.7)		.31		80 (37.0)	
	Chemotherapy		7 (9)		10 (7.4)		.70		17 (7.9)	
**Comorbidity, n (%)**							.20		
	None		18 (23)		19 (14.0)				37 (17.1)	
	1		15 (19)		35 (25.7)				50 (23.1)	
	≥2		45 (56)		78 (57.4)				123 (56.9)	
**Marital status,** ^b^ **n (%)**							.01		
	Partner		66 (83)		92 (67.6)				158 (73.1)	
	No partner		12 (15)		42 (30.9)				54 (25.0)	
**Educational level,** ^c^ **n (%)**							<.001		
	High		16 (20)		7 (5.1)				23 (10.6)	
	Intermediate		59 (74)		83 (61.0)				142 (65.7)	
	Low		5 (6)		42 (30.9)				47 (21.8)	
**Employed, n (%)**							.01		
	Yes		21 (28)		16 (11.8)				37 (17.1)	
	No		54 (72)		107 (78.7)				161 (74.5)	

^a^*P* values report comparisons between patients reporting disease-related Internet use and patients not reporting disease-related Internet use according to *t* tests and chi-square tests.

^b^Marital status: partner=married/living together, no partner=divorced/widowed/never married.

^c^Educational level: low=no/primary school, intermediate=lower general secondary education/vocational training, high=preuniversity education/high vocational training/university.

### Moderation of Disease-Related Internet Use

Four statistically significant moderation tests were found. Disease-related Internet use moderated the intervention effect on the amount of information received about the disease (*P*=.03), the amount of information received about medical tests (*P*=.01), the helpfulness of the information (*P*=.01), and how well patients understand their illness (*P*=.04). All other interaction terms were not significant.

**Figure 2:**
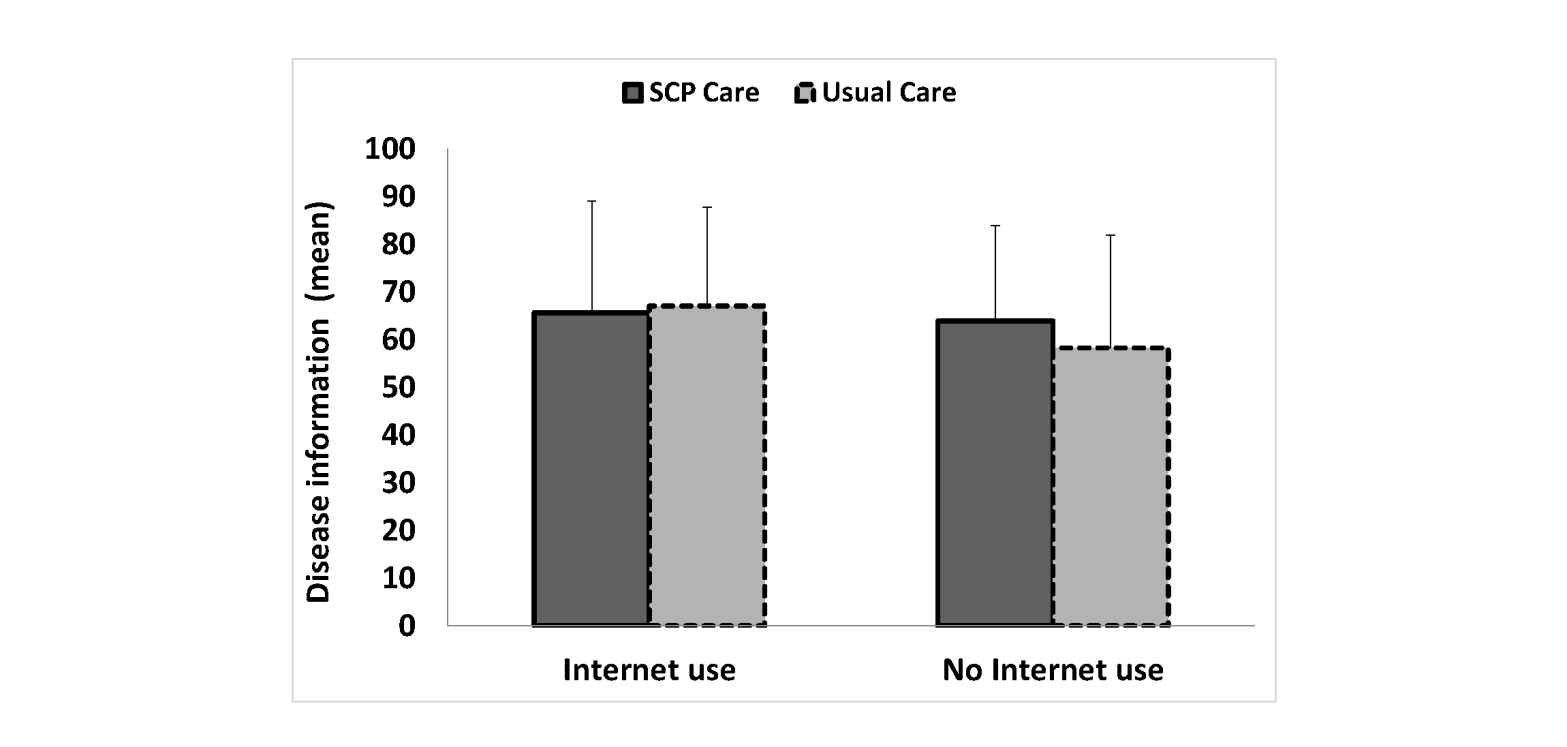
Patients’ reported amount of information received about their disease according to disease-related Internet use for the SCP care and the usual care arms. Crude means are reported. Error bars represent +1 SD. EORTC-QLQ-INFO25 scale ranges from 0-100 (higher scores reflect better perceived information received).

**Figure 3:**
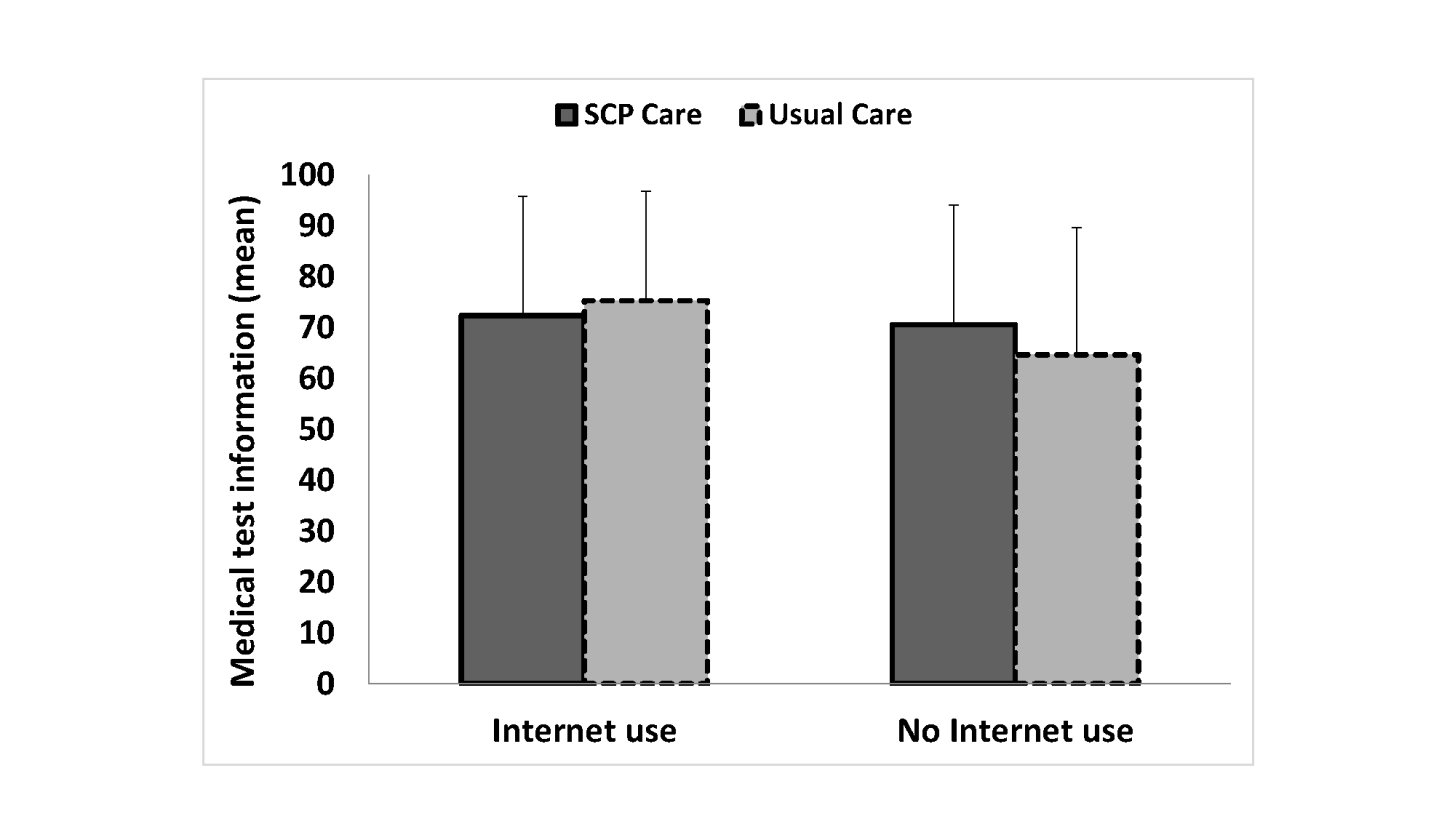
Patients’ reported amount of information received about their medical tests according to disease-related Internet use for the SCP care and the usual care arms. Crude means are reported. Error bars represent +1 SD. EORTC-QLQ-INFO25 scale ranges from 0-100 (higher scores reflect better perceived information received).

**Figure 4:**
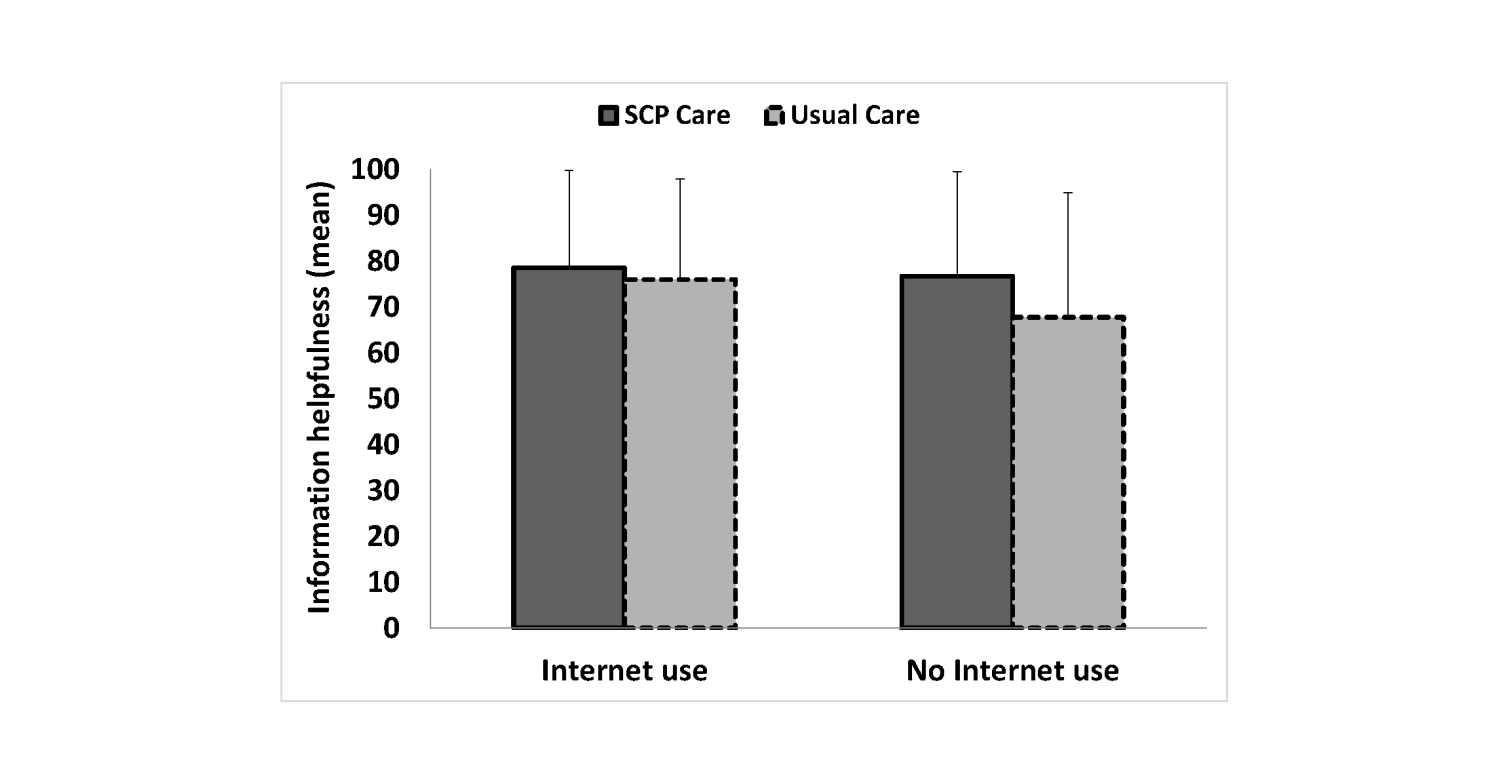
Patient-reported helpfulness of the information received according to disease-related Internet use for the SCP care arm and the usual care arm. Crude means are reported. Error bars represent +1 SD. EORTC-QLQ-INFO25 scale ranges from 0-100 (higher scores reflect better-perceived information received).

**Figure 5:**
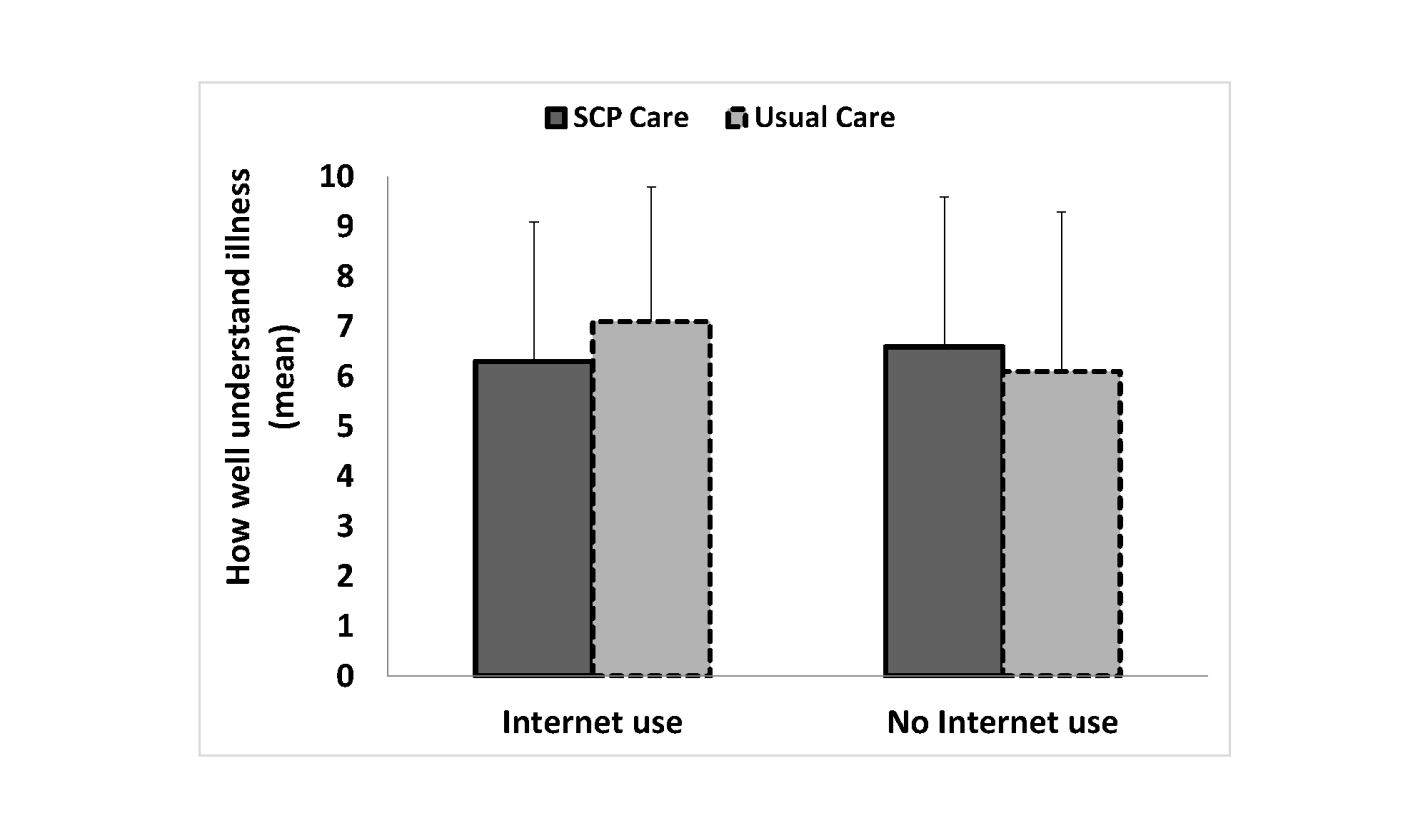
Patients’ reported understanding of their illness according to disease-related Internet use for the SCP care and the usual care arms. Crude means are reported. Error bars represent +1 SD. B-IPQ scale ranges from 1-10 (higher scores indicate more endorsement of that item).

Although the stratified analyses were all not statistically significant, it appeared that patients who did not seek disease-related information on the Internet may have benefitted from receiving an SCP because patients in the SCP care arm reported receiving more information about their disease (mean 63.9, SD 20.1 vs mean 58.3, SD 23.7) and medical tests (mean 70.6, SD 23.5 vs mean 64.7, SD 24.9), found the information more helpful (mean 76.7, SD 22.9 vs mean 67.8, SD 27.2), and understood their illness better (mean 6.6, SD 3.0 vs mean 6.1, SD 3.2) than patients in the usual care arm did (Table 4 and Figures 2-5). On the other hand, although the stratified analyses were all not statistically significant, it appeared that patients who did seek disease-related information on the Internet did not benefit from receiving an SCP because patients in the SCP care arm did not report receiving more information about their disease (mean 65.7, SD 23.4 vs mean 67.1, SD 20.7) and medical tests (mean 72.4, SD 23.5 vs mean 75.3, SD 21.6), did not find the information more helpful (mean 78.6, SD 21.2 vs mean 76.0, SD 22.0), and reported less understanding of their illness (mean 6.3, SD 2.8 vs mean 7.1, SD 2.7) than patients in the usual care arm did (Table 4 and Figures 2-5).

**Table 4:** Regression outcomes from the stratified analyses for the effect of SCP care on the outcomes according to disease-related Internet use.

Outcome^a^		SCP care, mean (SD)^b^	Usual care, mean (SD)^b^	Total, mean (SD)	Beta (95% CI)^c^	*P*
**Information disease** ^d^						
	Internet use	65.7 (23.4)	67.1 (20.7)	66.4 (22.1)	–1.36 (–12.7, 10.0)	.79
	No Internet use	63.9 (20.1)	58.3 (23.7)	61.4 (21.9)	5.51 (–3.9, 14.9)	.22
**Information medical tests** ^d^						
	Internet use	72.4 (23.5)	75.3 (21.6)	73.9 (22.6)	–3.83 (–13.5, 5.8)	.43
	No Internet use	70.6 (23.5)	64.7 (24.9)	68.0 (24.3)	4.87 (–3.3, 13.0)	.24
**Helpfulness information** ^d^						
	Internet use	78.6 (21.2)	76.0 (22.0)	77.3 (21.6)	1.13 (–7.4, 9.6)	.79
	No Internet use	76.7 (22.9)	67.8 (27.2)	72.9 (25.2)	6.89 (–1.6, 15.4)	.11
**How well understand illness** ^e^						
	Internet use	6.3 (2.8)	7.1 (2.7)	6.7 (2.8)	–0.98 (–2.11, 0.14)	.09
	No Internet use	6.6 (3.0)	6.1 (3.2)	6.3 (3.1)	0.30 (–0.73, 1.33)	.56

^a^Outcomes are presented only for the statistically significant interaction terms. Linear multilevel regression analyses were performed, adjusted for age, time since diagnosis, marital status, educational level, employment, comorbidities, stage, and treatment. For the models that did not converge, hospital was included as covariate instead of random intercept.

^b^Crude means and standard deviations are reported for SCP care and usual care.

^c^Unstandardized betas and 95% confidence intervals are reported for SCP care (ref=usual care).

^d^EORTC-QLQ-INFO25 scale range from 0-100: higher scores reflect better-perceived information received.

^e^B-IPQ scale range from 1-10: higher scores indicate more endorsement of that item.

## Discussion

The results of this secondary analysis of the ROGY Care trial suggest that paper-based SCPs appear to improve the amount of received information about the disease and medical tests, the helpfulness of the information, and the understanding of the illness for patients who do not search for information on the Internet themselves. In contrast, paper-based SCPs do not appear helpful for patients who already search for information on the Internet themselves. All other outcomes did not differ for patients who did or did not use the Internet to search for disease-related information.

### Patients Who Did Not Use the Internet to Search for Disease-Related Information

Nearly two-thirds of all patients in this study did not use the Internet to search for disease-related information. These patients were older, lower educated, and less often had a partner or a job than patients who did use the Internet to search for information about their cancer. This has consistently been found in previous studies [15,17,19] and has raised the concern that some patient groups do not equally benefit from the various resources available on the Internet [19]. Because educational level is an indicator for SES [39,40], patients with a higher SES search the Internet more for disease-related information than patients with a lower SES. This “digital divide” may pose a threat to equity in health care when important information can only be or best be accessed online [19]. Even today, a large number of cancer survivors do not have access to the potential benefits of the Internet. The results of this study suggest that paper-based SCPs may be a useful tool to empower this patient group by increasing the amount of information they receive about their disease and medical tests, the helpfulness of the information, and their understanding of their illness.

### Patients Who Did Use the Internet to Search for Disease-Related Information

A third of all patients in this study did use the Internet to search for disease-related information, which is consistent with previous studies [14-19]. The results of this study suggest that paper-based SCPs may not be of added value for this patient group. A possible explanation for this finding could be that these patients already benefit from accessing information on the Internet because using the Internet to obtain disease-related information has been associated with considerable benefits for cancer survivors [20]. Previous studies have found that cancer survivors who use the Internet to access disease-related information feel better informed [15], report receiving more information about their disease and medical tests [21], find the received information more helpful [21], communicate more effectively with their health care providers [22], and are more actively involved in decision making [23].

Surprisingly, the results of this study suggest that paper-based SCPs may actually even decrease patients’ understanding of their illness for those patients who search for disease-related information on the Internet. A possible explanation could be that patients who receive an SCP and also search for information on the Internet may find information on the Internet about their illness that conflicts with information within the SCP. This may confuse patients and may lower their understanding of the illness. Because these patients have access to more information, they may also be more aware of aspects of their illness that they do not (completely) understand (ie, the more you know, the more you realize how little you know). Future research needs to investigate why SCPs may not be helpful for patients who search for disease-related information on the Internet. Another possibility is that paper-based SCPs in their current form are not suitable for patients who search for disease-related information on the Internet. A possible way to increase the value of SCP care for patients who search for disease-related information on the Internet may be to provide these patients with access to a tailored online SCP instead of a paper-based SCP. Previous research showed that most patients who use the Internet prefer to get their information from reliable websites, such as their hospital’s website, and would like to have online access to their own medical file and test results [15]. Internet-based SCPs may be a useful way to support these patients in finding reliable information online that is tailored to their specific situation. The results of previous studies investigating cancer patients’ satisfaction with an Internet-based SCP tool seem promising [41-43]. Future research needs to examine whether dissemination of tailored online SCPs does have added value for patients who search for cancer-related information on the Internet.

### Considerations

It is important to take into consideration that this study was conducted in the Netherlands, a developed country where 95% of the population has access to the Internet at home [44]. Furthermore, only endometrial cancer patients were included in this study. In general, endometrial cancer patients have a lower educational level than patients with other types of cancer [45] and lower educational levels have been found to be strongly associated with lower Internet use [15,17,19]. In addition, men tend to use the Internet more often than women [15]. Consequently, the percentage of patients who used the Internet to search for disease-related information in this study may be an underestimation of the Internet use of cancer survivors in the Netherlands. A previous study conducted in the Netherlands in 2006 that included both male and female patients with different types of cancer found that 60% reported using the Internet by themselves [15].

Other effects of SCP care found in the ROGY Care trial [8], such as increased concerns about the illness, emotional impact, experienced symptoms, and health care utilization, did not differ for patients who did or did not use the Internet to search for disease-related information. This finding indicates that SCPs increase patients’ concerns, emotional impact, experienced symptoms, and health care utilization for both patients who do and do not search for disease-related information on the Internet. It is possible that certain aspects of the SCP that are not found on the Internet (eg, receiving information from the physician, receiving personalized information, and receiving information about additional care) lead to increased concerns, emotional impact, experienced symptoms, and health care utilization. However, it is important to consider that the ROGY Care trial was not originally powered to detect differences in moderation analyses or stratified analyses. Therefore, it is unclear whether insignificant outcomes in these analyses indicate that disease-related Internet use did not moderate these outcomes or that the power was merely too small to find the effects. On the other hand, this does make the moderation effects that were found in this study more convincing.

### Strengths and Limitations

A limitation of this study is that self-reported information provision and health care utilization were assessed, which makes it unclear how much information was actually provided and how much health care was actually used. In addition, Internet utilization was measured with a single dichotomous item. Consequently, this study can only make a distinction between patients who did or did not use the Internet to search for disease-related information. For instance, it remains unknown how many times patients searched the Internet, what they searched for (ie, did they use the Internet to search for similar topics as addressed in the SCP?), or what information they found. For future research, we recommend using a more elaborate measure of Internet utilization that is psychometrically tested.

Despite these limitations, this study provides important new insight into whether certain groups of patients may or may not benefit from paper-based SCPs in routine clinical practice. The pragmatic cluster randomized design, limited exclusion criteria, and high response rate improve the generalizability of the findings. However, there is not enough evidence to recommend that patients who search for information on the Internet should not receive a paper-based SCP. More research is needed to get a more nuanced understanding of these findings before health care providers can use the information to decide whether providing a paper-based SCP is of added value or not. In addition, future research needs to examine whether other patient characteristics could also possibly influence the impact of SCPs.

### Conclusions

The results of this secondary analyses of the ROGY Care trial suggest that paper-based SCPs may improve the amount of received information about the disease and medical tests, the helpfulness of the information, and the understanding of the illness for patients who do not search for information on the Internet themselves. In contrast, paper-based SCPs do not seem beneficial for patients who do search for disease-related information on the Internet. With the increasing importance of the Internet as a source of information for cancer survivors, future research needs to examine whether dissemination of tailored online SCPs may have added value for patients who use the Internet to obtain disease-related information.

## Appendix

Multimedia Appendix 1 Example of the ROGY Care SCP.

## Abbreviations

B-IPQ: Brief Illness Perception Questionnaire
EORTC: European Organisation for Research and Treatment of Cancer
FIGO: Federation of Gynecology and Obstetrics
IOM: Institute of Medicine
ROGY: Registrationsystem Oncological GYnecology
SCP: Survivorship Care Plan
SES: socioeconomic status
