# Supplementary material for: Paper-Based Survivorship Care Plans May be Less Helpful for Cancer Patients Who Search for Disease-Related Information on the Internet: Results of the Registrationsystem Oncological Gynecology (ROGY) Care Randomized Trial
Source: J Med Internet Res. 2016 Jul 8;18(7):e162. doi: 10.2196/jmir.4914 (PMC4958141; doi:10.2196/jmir.4914)
Supplement: Multimedia Appendix 1 [file jmir_v18i7e162_app1.pdf]

Zorgplan kanker

Persoonlijk zorgplan

Baarmoederkanker

Maxima Medisch Centrum, locatie Eindhoven

Ds Theodor Fliednerstr 1

5631 BM EINDHOVEN

T. 040-8888000

máxima medisch centrum

Uw persoonlijk zorgplan

Persoonsgevens

NAAM PATIENT:	Nicolaije
GEBOORTEDATUM:	23-11-1985

Contactgegevens

NAAM GYNAECOLOOG:	Kim Nicolaije
NAAM MEDISCH ONCOLOOG:	Creemers, G.J.
NAAM RADIOTHERAPEUT:	Lybeert, M.L.M.
DATUM START ZORGPLAN:	04-05-2011
DATUM ZORGPLAN BIJ HEROVERWEGING NAZORG:	04-05-2011

Voor u ligt uw persoonlijk zorgplan. Dit zorgplan bevat infomatie die specifiek op uw situatie van toepassing is. Het geeft u een overzicht van uw diagnose, behandeling en nazorgtraject. Verder geeft het u informatie over de mogelijke korte en lange termijn gevolgen van uw behandeling. Uw zorgplan kan in de loop van de tijd worden aangepast. U ontvangt daarom steeds een nieuwe versie van uw zorgplan.

Elke patiënt zal andere problemen ervaren in de tijd na de behandeling. Dit plan heeft niet tot doel om u ongerust te maken, maar wil u juist bewust maken van het feit dat u mogelijk nog lange tijd na uw behandeling hinder kunt ondervinden van uw ziekte.

Het is belangrijk dat u uw gezondheid in de gaten houdt en op tijd stappen onderneemt bij aanhoudende problemen. U kunt in uw zorgplan lezen bij welke klachten u in ieder geval contact op moet nemen, en bij wie u dan terecht kunt. Dit zorgplan is een persoonlijk document. Wij raden u daarom aan om er zorgvuldig mee om te gaan.

Diagnose

Op 04-05-2011 is bij u de diagnose baarmoederkanker gesteld.

In de tabel hieronder ziet u een overzicht van uw diagnose, met daaronder uitleg bij de diagnose. Op de volgende pagina staan afbeeldingen ter verduidelijking. Als u iets niet helemaal begrijpt of als u verdere uitleg nodig heeft, dan kunt u uw arts vragen om meer informatie.

Uw diagnose

TNM-stadium:	T1 N0 M0
FIGO-stadium:	IIIC
Gradering:	goed
Histologie:	mucineus adenocarcinoom

Stadium-indeling

Het stadium geeft met behulp van cijfers aan hoe ver de ziekte is voortgeschreden. In het algemeen geldt: hoe hoger het cijfer, hoe ernstiger de situatie is.

Het **TNM-stadium** wordt bepaald aan de hand van:

- de grootte van de tumor en de mate van doorgroei in het omringende weefsel; dit wordt aangeduid met de 'T' van tumor.
- de aanwezigheid van uitzaaiingen in de lymfeklieren; dit wordt aangeduid met de 'N' van 'node' (= Engels voor lymfeklier).
- de aanwezigheid van uitzaaiingen op afstand; deze wordt aangeduid met de 'M' van metastase.

De **FIGO-stadiumindeling** onderscheidt 4 stadia:

- **Stadium I:** de tumor is beperkt tot het baarmoederlichaam. Er wordt gekeken of de tumor is doorgegroeid in de spierlaag van de baarmoeder. Men maakt hierbij onderscheid tussen doorgroei in het oppervlakkige of diepere deel van de spierlaag.

- **Stadium II:** de tumor is doorgegroeid tot in de baarmoederhals, maar niet buiten de baarmoeder.

- **Stadium III:** de tumor is doorgegroeid buiten de baarmoeder, maar binnen het kleine bekken. Dat wil zeggen: in de directe omgeving van de baarmoeder, bijvoorbeeld naar de eierstokken, de vagina of de lymfeklieren in de buik.

- **Stadium IV:** de tumor is doorgegroeid buiten het kleine bekken of naar de blaas of de endeldarm en/of er zijn uitzaaiingen ergens anders in de buik. Ook bij uitzaaiingen van baarmoederkanker in andere organen, bijvoorbeeld in de longen of de botten, spreekt men van stadium IV.

Gradering

Gradering is de mate van kwaadaardigheid van de tumor. Deze wordt ingedeeld in 'goed', 'matig' of 'slecht'. Hoe 'slechter' de gradering, hoe kwaadaardiger de tumor en hoe sneller deze groeit.

Hieronder ziet u op de linker-afbeelding de inwendige vrouwelijke geslachtsorganen afgebeeld. Op de rechter-afbeelding ziet u uw FIGO-stadium, stadium IIIC, afgebeeld.

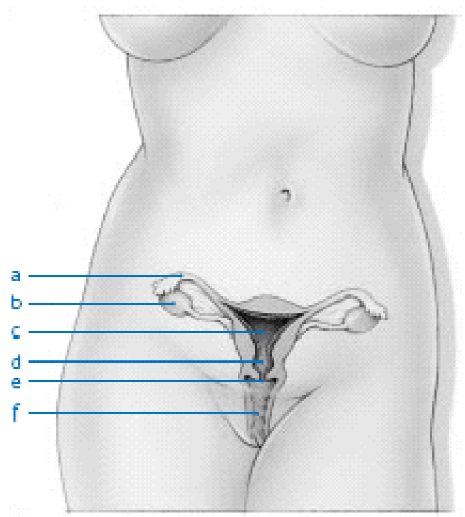

3.
De inwendige vrouwelijke geslachtsorganen

a. eileider
b. eierstok
c. baarmoederlichaam
d. baarmoederhals
e. baarmoedermond
f. vagina of schede

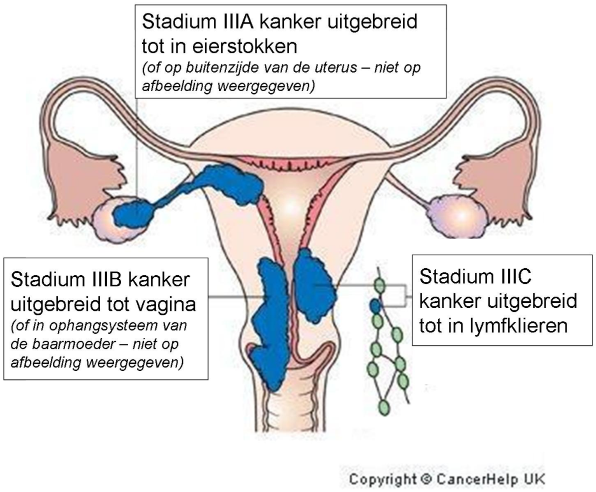

Behandeling

U bent behandeld voor baarmoederkanker. Uw behandeling bestond uit chirurgie waarna chemo-radiatie waarna chemotherapie.

- hormoontherapie
- debulking
- brachy, uitwendige radiotherapie
- mono chemotherapie met taxane, platinum bevattende stoffen

Hieronder vindt u een overzicht van de behandeling(en) die u heeft ondergaan:

	START-EINDDATUM	LOCATIE	BEHANDELAAR	BEHANDELING
hormoontherapie	04-05-2011		Boll, D. / Creemers, G.J.	-
chirurgisch	00-00-9999	Maxima Medisch Centrum, locatie Eindhoven	Boss, E.	debulking
radiotherapie	00-00-9999	Catharina-ziekenhuis, Radiotherapie	Lybeert, M.L.M.	brachy, uitwendige radiotherapie
chemotherapie	00-00-9999	Catharina-ziekenhuis	Creemers, G.J.	mono chemotherapie met taxane, platinum bevattende stoffen

Bij het opstellen van een behandelplan wordt altijd goed voor ogen gehouden wat het doel van de behandeling is. Het doel van uw behandelig is curatief. Een curatieve behandeling is erop gericht om iemand te genezen.

Mogelijke gevolgen van de behandeling op korte termijn

Meest voorkomende bijwerkingen van een operatie

Een operatie is de meest voorkomende behandeling bij baarmoederkanker. De buik wordt geopend met een snee die loopt van boven de navel tot aan het schaambeen. Meestal verwijdert de gynaecoloog de baarmoeder, de beide eierstokken en het grote inwendige vetschort (omentum majus).

Net zoals bij elke operatie bestaat er een kans op wondinfectie, stoornissen in de wondgenezing, trombose, lymfoedeem (opgezwollen benen door vochtophoping), blaasontsteking en het moeilijk op gang komen van de darmen. Dit gebeurt meestal in de eerste dagen tot weken na de ingreep. Een dergelijke bijwerking langer na de operatie is zeldzaam.

Colostoma

Bij een stoma van de dikke darm, een colostoma, is de ontlasting vaak van gewone dikte. De stoma geeft meestal eenmaal per dag veel ontlasting en mogelijk daarnaast verdeeld over de dag kleine beetjes. Een stomaverpleegkundige informeert u over de bestaande opvangmaterialen en de verzorging van de stoma.

Patiënten met een colostoma op het laatste deel van de dikke darm kunnen, na toestemming van hun arts, hun darmen legen door te 'spoelen'. Dit is te vergelijken met een klysma. Door water in te brengen wordt de dikke darm geactiveerd en leegt zich na enige tijd. Daardoor bent u langere tijd ontlastingvrij. Voor alle zekerheid moet de stoma wel worden afgedekt.

Ileostoma

Patiënten die een stoma van de dunne darm krijgen, een ileostoma, hebben dunnere ontlasting. Bij een ileostoma komt de ontlasting vrijwel gedurende de hele dag, maar vooral na de warme maaltijd.

Laparoscopie

Dit onderzoek wordt ook wel een kijkoperatie genoemd. Het is een van de mogelijkheden om definitief vast te stellen of er sprake is van baarmoederkanker. Laparoscopie wordt soms gedaan om te bepalen in welke mate de ziekte zich heeft uitgebreid.

Het onderzoek wordt uitgevoerd met een speciale kijkbuis, een laparoscoop, waarmee de buikholte kan worden bekeken. Ook kunnen buikvocht en kleine stukjes weefsel worden weggenomen (biopsie) voor nader onderzoek.

Laparoscopie vindt plaats onder algehele narcose. De laparoscoop wordt ingebracht via een kleine snee bij de navel of boven in de buik. Ter hoogte van het schaambeen maakt de gynaecoloog een tweede opening voor het inbrengen van instrumenten die nodig zijn voor de biopsie.

De gynaecoloog bekijkt tijdens het onderzoek de baarmoeder, eierstokken en eileiders. Ook kan hij zich een beeld vormen van het oppervlak van andere organen in de buik, zoals de blaas, darmen, maag, lever en milt.

Meest voorkomende bijwerkingen van radiotherapie

Bestraling (radiotherapie) is een plaatselijke behandeling met als doel de kankercellen te vernietigen, terwijl de gezonde cellen zo veel mogelijk gespaard blijven. Kankercellen verdragen straling slechter dan gezonde cellen en herstellen zich er minder goed van. Gezonde cellen herstellen zich over het algemeen wel.

Bestraling zonder een voorafgaande operatie wordt alleen toegepast als een operatie niet goed mogelijk is door de uitgebreidheid van de baarmoederkanker of doordat de gezondheidstoestand geen operatie toelaat. Bestraling kan uitwendig, inwendig of als combinatie worden toegepast.

Inwendige radiotherapie

Bij inwendige bestraling (brachytherapie, brachy = Grieks voor 'dichtbij') wordt radioactief materiaal in de baarmoeder en/of het bovenste deel van de vagina geplaatst en vindt bestraling van binnenuit plaats. Hiervoor brengt de arts holle buisjes (bronhouders) in. Dit gebeurt onder plaatselijke verdoving.

Tijdens de inwendige bestraling verblijft u, vanwege de straling, in een kamer met speciale voorzieningen. Daar wordt u aangesloten op een 'afterloading apparaat'. Dit apparaat brengt radioactiviteit over naar de bronhouders. De radiotherapeut berekent nauwkeurig hoeveel straling u nodig heeft. Tijdens de behandeling wordt via een blaaskatheter de urine afgevoerd. Als de bestraling klaar is, wordt het afterloading apparaat losgekoppeld en worden de bronhouders verwijderd. U bent daarna vrij van straling.

Er kunnen klachten ontstaan na inwendige bestraling. Soms is het plassen enkele dagen wat gevoelig, ontstaat vaginale afscheiding, bloedverlies of diarree. Omdat de inwendige bestraling tijdens of kort na de uitwendige bestraling plaatsvindt, kunt u ook last hebben van de bijwerkingen van de uitwendige bestraling.
Vooral bij een combinatie van uitwendige en inwendige bestraling kan het bovenste deel van de vagina stugger en droger worden. Dit kan seksuele activiteit bemoeilijken. Uw arts zal u adviezen geven om dit zo veel mogelijk te voorkomen.

Uitwendige radiotherapie

De straling komt uit een bestralingstoestel (lineaire versneller). Het te behandelen gebied wordt van buitenaf - door de huid heen - bestraald. Om het te bestralen gebied nauwkeurig te bepalen, wordt vooraf een CT-scan gemaakt. De radiotherapeut of radiotherapeutisch laborant zorgt ervoor dat de stralenbundel nauwkeurig wordt gericht en dat het omliggende, gezonde weefsel zo veel mogelijk buiten het te bestralen gebied blijft. Over het algemeen duurt een bestralingsbehandeling een aantal weken en heeft vier tot vijf maal per week plaats. In die periode krijgt u per keer gedurende een aantal minuten een dosis straling. Voor uitwendige bestraling is meestal geen opname in het ziekenhuis nodig. Bij patiënten die uitwendige bestraling krijgen als adjuvante behandeling, begint de bestralingsserie enkele weken na de operatie.

Bestraling beschadigt niet alleen kankercellen, maar ook gezonde cellen in het bestraalde gebied. Daardoor kunt u met een aantal bijwerkingen te maken krijgen:
- Omdat bij bestraling van de onderbuik ook de darmen straling krijgen, kunnen sommige vrouwen last krijgen van buikkrampen en een verandering van hun normale ontlastingpatroon. De ontlasting kan slijmerig zijn en gepaard gaan met wat bloedverlies.
- Omdat bij bestraling van de onderbuik ook de blaas straling krijgt, moeten sommige vrouwen vaker plassen en is er een grotere kans op blaasontsteking.
- Bij sommige patiënten ontstaat een rode of donker verkleurde huid op de bestraalde plek.
- Over het algemeen hebben patiënten gedurende de bestralingsperiode last van vermoeidheid.

De meeste klachten verdwijnen meestal enkele weken na afloop van de behandeling. Sommige mensen merken echter nog lang na hun behandeling dat zij eerder vermoeid zijn dan voor hun ziekte. Ook kunnen klachten soms langer aanhouden, zoals de eerdergenoemde darm- en blaasklachten. Uw arts kan hiervoor medicijnen voorschrijven.

Meest voorkomende bijwerkingen van hormonale therapie

Hormonen zijn stoffen die ons lichaam zelf maakt. Deze worden uitgescheiden in het bloed en beïnvloeden bepaalde processen of organen in ons lichaam. Een belangrijke groep hormonen zijn de geslachtshormonen.

Baarmoederkanker kan gevoelig zijn voor het vrouwelijk geslachtshormoon progesteron. Hormonale therapie is op die gevoeligheid gebaseerd. Door het gebruik van progesteron (vaak in hoge dosering) kan het ontstaan en de woekering van de kankercellen (tijdelijk) worden stopgezet.

Bij hormonale therapie kunnen - onder meer afhankelijk van de voorgeschreven dosis - de volgende bijwerkingen optreden:

- een lichte toename van het lichaamsgewicht (1 tot 2 kilo)
- extra belasting van het hart en de bloedvaten omdat het lichaam meer vocht vasthoudt
- misselijkheid (in het begin van de behandeling)

Meest voorkomende bijwerkingen van chemotherapie

Chemotherapie is de behandeling van kanker met celdodende of celdelingremmende medicijnen: cytostatica. Er zijn verschillende soorten cytostatica, elk met een eigen werking. De medicijnen kunnen op verschillende manieren worden toegediend, bijvoorbeeld per infuus of injectie.

Soms worden zij met behulp van een buikkatheter (een dunne slang) rechtstreeks in de buikholte toegediend. Via het bloed verspreiden zij zich door uw lichaam en kunnen op vrijwel alle plaatsen kankercellen bereiken.

Of u last krijgt van bijwerkingen hangt onder meer af van de soorten en hoeveelheden cytostatica die u krijgt.
Cytostatica, in uw geval taxane en platinum bevattende stoffen, tasten naast kankercellen ook gezonde cellen aan. Daardoor kunnen onaangename bijwerkingen optreden, zoals:

Beenmerg - bloedarmoede

Bloedarmoede is een tekort aan rode bloedcellen (erytrocyten) en wordt ook wel anemie genoemd.

U hebt een verhoogde kans op:
- kortademigheid en vermoeidheid, zelfs als u maar heel weinig hebt gedaan
- het zwart voor de ogen zien bij opstaan uit bed of stoel
- bleekheid, lusteloosheid
- duizeligheid, hoofdpijn
- niet goed kunnen slapen of niet goed kunnen concentreren
- hartklachten of hartkloppingen
- koud gevoel, transpireren

Beenmerg - leukopenie

Leukopenie is een tekort aan witte bloedlichaampjes (leukocyten) in uw bloed. Witte bloedlichaampjes zorgen voor afweer tegen infecties. Bacteriën of ziekten die voor de gezonde mens weinig gevaar opleveren, kunnen bij u –omdat u wat kwetsbaarder bent- tot heftige reacties leiden met hoge koorts. Ongeveer tussen de 10^{e} en de 15^{e} dag na het starten van de kuur hebt u de minste witte bloedlichaampjes. Dit heet een dipperiode. Daar kunt niets tegen doen. Er is wel een aantal maatregelen dat u kunt nemen om de kans op infecties in zo'n periode zoveel mogelijk tegen te gaan. Een infectie is onder andere te herkennen aan een lichaamstemperatuur van 38,5 ºC of hoger, soms met koude rillingen.

Afhankelijk van de plaats waar de infectie zit, kunnen dan de volgende klachten ontstaan:
- slijm ophoesten
- pijn bij het plassen

- troebele urine
- vaker plassen
- pijnlijke plekken in de mond of pijn bij het slikken
- buikpijn
- diarree

Beenmerg - trombocytopenie

Trombocytopenie is een tekort aan bloedplaatjes (trombocyten) in het bloed. Bloedplaatjes spelen een belangrijke rol bij de bloedstolling. Door een daling van het aantal bloedplaatjes is het bloed dunner en stolt het minder snel.

U hebt een verhoogde kans op:
- een neusbloeding die langer aanhoudt
- blauwe plekken
- bloed bij plassen
- bloed bij hoesten
- bloedend tandvlees
- puntvormige bloedinkjes in de huid
- bloed bij braken
- bloed in de ontlasting
- bij vrouwen kan de menstruatie anders zijn

In uitzonderlijke gevallen treden er spontane bloedingen op.

Bloeddruk - verlaagde

Als u lage bloeddruk heeft, zult u dat het vaakst merken bij plotseling opstaan. U voelt zich slap en duizelig. Soms kunt u zelfs flauwvallen.

Darmen - diarree

Diarree is een waterige dunne ontlasting die meer dan 4 keer per dag voorkomt. De opname van vocht en voedingsstoffen in uw darmen is dan verstoord. Dat komt door irritatie van het slijmvlies van de darm en door een verandering in de stofwisseling van de dunne darm. Bij diarree worden voedingsstoffen in de darmen minder goed opgenomen.

U kunt last hebben van de volgende klachten:
- buikpijn/buikkrampen
- vaak aandrang om het toilet te bezoeken
- dunne ontlasting

Darmen - verstopping

Klachten bij verstopping zijn:
- harde en droge ontlasting
- persen bij stoelgang
- opgezette buik
- buikpijn/darmkrampen
- verminderde eetlust door een vol gevoel

Gehoorafwijkingen

Door de behandeling kan het zijn dat u slechter hoort dan u gewend bent en u kunt oorsuizingen hebben. Met behulp van een audiogram (gehooronderzoek) worden de klachten onderzocht (gemeten) en wordt geprobeerd gehoorverlies in een vroeg stadium te voorkomen of in ieder geval te beperken.

Grieperig gevoel, spierpijn

Door de behandeling kunt u een grieperig gevoel krijgen, met verschijnselen van:
- algehele malaise
- koorts
- hoofdpijn
- verminderde eetlust
- spierpijn en pijn in de botten

Het grieperig gevoel is meestal van korte duur. Het begint enige uren na de toediening van de medicijnen, houdt 1 tot 2 dagen aan en verdwijnt meestal weer spontaan.

Haar - kaalheid

U kunt zelf niets doen om haaruitval te voorkomen. Voorkomende verschijnselen kunnen zijn:
- behalve hoofdhaar kunnen ook wenkbrauwen, wimpers, oksel-, lichaams- en schaamharen uitvallen
- wanneer uw haar weer aangroeit, kan het anders zijn dan uw oorspronkelijke haar was, zoals: de kleur, het haar kan sluiker zijn of juist meer slag hebben; meestal is dit tijdelijk
- haaruitval begint meestal enkele weken na toediening van medicijnen
- uw hoofdhuid kan gevoelige of pijnlijke aanvoelen
- uw hoofdhaar zal eerder en sneller uitvallen dan uw lichaamshaar
- uitvallen van veel haar tegelijk vinden de meeste mensen heel vervelend; het haar kort knippen kan dan een oplossing zijn.

Leverfunctiestoornis

Door de behandeling kan de leverfunctie verstoord raken. Stoornissen van de leverfunctie zijn vaak te zien aan afwijkingen in het bloed. Daar zult u in eerste instantie niet veel van merken. Pas bij ernstige leverfunctiestoornissen kunt u klachten krijgen als vermoeidheid, complete malaise of u krijgt geelzucht.
Als er leverfunctiestoornissen optreden, kunnen die het verloop van de behandeling veranderen. U krijgt bijvoorbeeld een lagere dosis toegediend of de behandelend arts schrijft een ander middel voor.
Leverfunctiestoornissen kunnen ontstaan na toediening van de eerste medicijnen.

Misselijkheid en braken

Door de behandeling kunt u last krijgen van misselijkheid en braken.
U kunt de volgende klachten krijgen:
- kokhalzen en braken
- weinig of geen eetlust
- maagklachten, zoals een vol gevoel of pijn
- buikpijn of -krampen, opgezette buik, rommelingen in de buik
- dorst

Medicijnen kunnen misselijkheid en braken verminderen of voorkomen. Het is belangrijk dat u de medicijnen altijd inneemt zoals u met uw behandelend arts hebt besproken. Houdt u aan de vaste tijden om de medicijnen in te nemen, ook als u niet misselijk bent.

Mond en lippen (pijnlijke)

Door de behandeling kunt u last krijgen van irritatie, beschadiging of ontsteking van het mondslijmvlies en u kunt last krijgen van tandbederf.
Klachten waaraan u merkt dat het mondslijmvlies is veranderd zijn:
- droge mond en brandend gevoel
- gevoeligheid voor de temperatuur van eten en drinken
- gevoeligheid bij het eten of drinken van zure of gekruide spijzen en dranken

- snel bloedend tandvlees
- slechte adem

Het is belangrijk om bij deze problemen uw mondholte goed te verzorgen.

Nierfunctiestoornis

Door de behandeling kan er een beschadiging aan het nierweefsel ontstaan waardoor de functie van de nier achteruit gaat.
Om uw nieren zoveel mogelijk te beschermen, krijgt u tijdens de behandeling veel vocht toegediend via een infuus.

Overgevoeligheid (allergie)

De behandeling kan een allergische reactie geven. Meestal ontstaan allergische klachten direct na het innemen of toediening van medicijnen, soms pas na enkele uren. Een allergische reactie begint vaak met:

- roodheid en huiduitslag, soms met jeuk over het hele lichaam
- kuchen
- onrust

Wat later kunnen de volgende verschijnselen optreden:

- bleek zien
- gezwollen oogleden en een opgezet gezicht
- beklemmend gevoel op de borst
- duizeligheid en bloeddrukdaling
- rillen
- misselijkheid en darmkrampen
- kortademigheid
- gevoel van onrust

Smaak (verandering of vermindering)

Door de behandeling kan uw smaak veranderen of verminderen. Wat u proeft of hoe iets smaakt verandert door verhoging of verlaging van de zogenaamde ‘smaakdrempels’ De uitgesproken smaken zoals zoet, zout, zuur of bitter worden anders geproefd. Een bittere smaak (‘metalig, gallig’) kan overheersen. Ook uw reuk zal misschien veranderen. Er kan een ongevoeligheid voor geuren ontstaan (u ruikt veel minder of niks) of juist een overgevoeligheid voor geuren (u ruikt alles sterker) die beide niet overeenkomen met de smaak. Uw smaakvoorkeur is niet meer hetzelfde.

Zenuwstelsel - perifeer

Als zenuwencellen zijn beschadigd, zullen prikkelingsverschijnselen optreden. Deze klachten zijn het ergst meteen na de behandeling, maar ze verminderen in de weken daarna. Het kan ook zijn dat de klachten pas enkele dagen na de behandeling beginnen. Ze verdwijnen meestal binnen enkele maanden; soms zijn blijvend.

De volgende klachten kunnen zich voordoen:

- minder gevoel in handen en/of voeten, vingers en/of tenen
- tintelingen in handen en/of voeten, vingers en/of tenen
- een branderig of juist slapend gevoel in handen en/of voeten, vingers en/of tenen
- minder kracht in armen of benen
- pijnklachten van handen, voeten en huid bij koude omstandigheden

Mogelijke gevolgen van de behandeling op lange termijn

Na uw behandeling zult u niet altijd meer direct in contact staan met uw behandelend arts. U zult echter nog wel te maken krijgen met de lange termijn gevolgen van uw behandeling. Soms is het heel lastig voor u om lichaamssignalen te interpreteren. Hieronder vindt u een overzicht van de mogelijke effecten van uw behandeling:

Vermoeidheid

Vermoeidheid kan ontstaan door kanker en/of de behandeling van kanker. Steeds meer mensen geven aan hiervan last te hebben. Sommigen krijgen enige tijd na de behandeling nog last van (extreme) vermoeidheid. De vermoeidheid kan lang aanhouden. Wanneer de ziekte vergevorderd is, kan de vermoeidheid ook te maken hebben met het voortschrijdende ziekteproces.

Vermoeidheid na kanker is niet bij iedereen hetzelfde. Misschien heeft u er in een bepaalde periode veel last van, afgewisseld door weken of maanden met meer energie. Of misschien voelt het bij u eerder aan als een constant gebrek aan energie. Toch zijn er enkele bijzonderheden die veel mensen noemen en waardoor vermoeidheid na kanker zich onderscheidt van gewone vermoeidheid.

De volgende kenmerken worden door veel mensen genoemd:

- Het is er plotseling, zonder waarschuwing, en meestal niet als gevolg van een inspanning.
- De vermoeidheid wordt vaak als extreem ervaren en lijkt op uitputting.
- De herstelperiode is langer dan na een 'normale' vermoeidheid.

Een belangrijk verschil tussen vermoeidheid na kanker en gewone vermoeidheid is dat de ernst van de vermoeidheid in geen enkele verhouding staat tot de verrichte activiteit of inspanning. De vermoeidheid lijkt uit het niets op te duiken en overvalt de persoon in kwestie. En dan ook nog vaak op de vreemdste momenten van de dag, bijvoorbeeld vlak na het opstaan, zelfs na een goede nachtrust.

Veel mensen ervaren de vermoeidheid als extreem. Er kunnen diverse lichamelijke klachten mee gepaard gaan, zoals hoofdpijn, duizeligheid, misselijkheid, keelpijn, pijn in de ogen, het koud hebben, klappertanden en rillen, jeuken of gloeien van de huid en grieperig en koortsig zijn.

Benen en armen kunnen loodzwaar worden of aanvoelen als rubber. Misschien heeft u last van een verminderend coördinatiegevoel: u struikelt en laat dingen uit uw handen vallen. Of heeft u te kampen met concentratieverlies: u vergeet dingen en kunt geen boek of krant lezen omdat u de informatie niet meer goed kunt opnemen. De meeste mensen zeggen ook slecht tegen stress te kunnen.

Onvruchtbaarheid

In zeldzame gevallen komt baarmoederkanker voor bij vrouwen onder de 40 jaar. Na de behandeling van baarmoederkanker waarbij de baarmoeder en de eierstokken zijn verwijderd, worden vrouwen onvruchtbaar. Wanneer u nog kinderen had willen krijgen, kunt u het hierdoor extra moeilijk hebben.

Overgang

Voor vrouwen die nog niet in de overgang waren, betekent verwijdering van de baarmoeder dat er een eind komt aan de menstruatie. Daarnaast betekent het verwijderen van de eierstokken dat de productie van bepaalde geslachtshormonen wordt beëindigd. Hierdoor komt u vervroegd in de

overgang. Net als de natuurlijke overgang kan dit verschijnselen veroorzaken als 'opvliegers', overmatige transpiratie en het afwisselend warm en koud hebben. Het plotseling wegvallen van de hormoonproductie kan worden opgevangen door hormoonvervangende medicijnen. Deze zijn ook van belang in verband met de mogelijke gevolgen van een verstoorde hormoonproductie op de lange duur, zoals botontkalking.

Als het echter om een hormoongevoelige tumor ging, kunnen hormonen eventueel achtergebleven kankercellen juist tot groei aanzetten. Daarom is het belangrijk eerst met de behandelend specialist te overleggen of een hormoonpreparaat gebruikt mag worden om de overgangsverschijnselen tegen te gaan.

Plasproblemen

Soms is het functioneren van de blaas na de operatie wat veranderd. Het kan zijn dat u af en toe wat urine verliest (incontinentie), vaker op de dag moet plassen, of aandrang heeft om te plassen. Het kan ook zijn dat u de eerste tijd (weken tot maanden) niet goed kunt uitplassen.

Sommige vrouwen hebben na de operatie moeite met het ophouden van de urine. Dat is het geval als bij de operatie kleine zenuwen van de blaas zijn beschadigd. Dat is niet altijd te voorkomen. Normaal geven deze zenuwen een signaal dat u moet plassen. Als deze niet meer functioneren, raakt de blaas te vol. U verliest dan zonder voorafgaande aankondiging ineens urine. De eerste maanden na de operatie is het daarom goed om op geregelde tijden te plassen. Meestal keert het signaal dat u moet plassen na enige tijd geleidelijk terug.

Emoties

Misschien schrikt u van uw eigen emoties. U herkent uzelf niet meer. Emoties als boosheid, paniek, wanhoop, angst of machteloosheid horen er allemaal bij. Het zijn heel normale reacties op een ingrijpende gebeurtenis.

Het kan moeilijk zijn om de diagnose direct onder ogen te zien. Voor sommige mensen zijn de emoties te hevig om in één keer toe te laten. Dat is heel begrijpelijk. Het lijkt wel een golf die je meesleurt. Er is niets op tegen om de werkelijkheid mondjesmaat tot u door te laten dringen. Het is een manier om uzelf in bescherming te nemen, om uzelf tijd te gunnen om aan het idee te wennen.

U kunt kwaad worden om wat u is overkomen. Het is oneerlijk dat dit lot net ú treft. Die boosheid richt zich soms op anderen. Bijvoorbeeld op uw partner. Juist mensen die u het dierbaarst zijn, krijgen vaak het meest te verduren. Begrijpelijk, maar niet redelijk. Probeer er op rustiger momenten met elkaar over te praten.

Kwaadheid kan een manier zijn om greep te krijgen op de situatie. Soms helpt het om uw gevoel nader onder de loep te nemen: ben ik echt boos, of misschien bang of verdrietig? Kwaad zijn lijkt soms makkelijker te zijn dan angstig of verdrietig. Hoe vreemd het misschien ook klinkt, als u angstig en verdrietig van uzelf 'mag' zijn, worden deze gevoelens mogelijk minder grimmig en heeft u er misschien minder last van.

Leren leven met kanker, je aanpassen aan een nieuwe situatie, kost tijd. Veel meer tijd dan de meeste mensen denken. Het is niet vreemd als u na een poosje merkt dat u zich somber, lusteloos of prikkelbaar voelt. Schrik niet als u het helemaal niet meer ziet zitten. Het hoort bij deze moeilijke periode. Na een poosje klaart uw stemming meestal weer op en merkt u dat het beter gaat.

Soms gebeurt het dat het verdriet van nu herinneringen aan nare gebeurtenissen uit het verleden losmaakt, de dood van een van uw ouders bijvoorbeeld of het stuklopen van een relatie. Wees daarop bedacht. Zoek professionele hulp als het u te veel wordt.

Het steeds beter leren leven met kanker gaat wel met vallen en opstaan. Vaak wisselen perioden waarin iemand heel erg bezig is met zijn ziekte zich af met tijden waarin het lukt om het gewone leven weer op te pakken.

Intimiteit en seksualiteit

Na de behandeling van baarmoederkanker wordt, in verband met de wondgenezing, meestal geadviseerd te wachten met het hebben van geslachtsgemeenschap tot na de eerste poliklinische controle, ongeveer zes weken na ontslag uit het ziekenhuis. In medisch opzicht zijn er geen bezwaren tegen seksuele opwinding, masturberen of het krijgen van een orgasme (klaarkomen). Voorop staat dat u voor uzelf moet bepalen wanneer u aan vrijen toe bent en op welke wijze u dat wilt. Het is belangrijk om dit met uw partner te bespreken. Geborgenheid, warmte en lichamelijk dicht bij elkaar zijn, zijn voor u wellicht belangrijker dan seksuele opwinding.

Door de behandeling kan een tekort aan geslachtshormonen ontstaan, waardoor de zin in vrijen is afgenomen. Sommige vrouwen doen er langer over om seksueel opgewonden te raken omdat de prikkeling vanuit de buik is afgenomen of veranderd. Ook streling van de borsten leidt soms tot minder opwinding dan voorheen.

Bij veel vrouwen bij wie de baarmoeder is verwijderd verandert het orgasme (klaarkomen). Dat geldt vooral voor vrouwen die bij het orgasme altijd hevige samentrekkingen van en rond de baarmoeder voelden. Sommige vrouwen ervaren het wegvallen van dit gevoel alleen vlak na de operatie, voor anderen is het een blijvend gemis. Er zijn ook vrouwen die deze samentrekkingen blijven voelen, ondanks de operatie. Voor vrouwen die het orgasme vooral in de buurt van de kittelaar (clitoris) en de binnenkant van de vagina voelden, zal het klaarkomen na de operatie meestal niet veel veranderen.

Als gevolg van de behandeling wordt tijdens seksuele opwinding de vagina bij sommige vrouwen minder vochtig. Daardoor kan geslachtsgemeenschap pijnlijk zijn. Meestal is een glijmiddel een goede oplossing. Glijmiddelen zijn bij de apotheek of bij de drogist verkrijgbaar.

In de loop van de maanden na de (inwendige) bestraling is het mogelijk dat het bovenste deel van de vagina stugger, droger en wat korter wordt. Om dit tegen te gaan, kan het verstandig zijn om, zolang u nog niet regelmatig geslachtsgemeenschap heeft, hulpmiddelen te gebruiken om de vagina beetje bij beetje op te rekken. Uw arts kan u hierover meer informatie geven. Soms ook is een glijmiddel een goede oplossing.

Wanneer er als gevolg van de behandeling seksuele problemen ontstaan, kunnen deze ook psychisch van aard zijn. Uw beleving van seksualiteit en uw gevoel van vrouw-zijn kan door de behandeling veranderd zijn. De ene vrouw ervaart dat sterker dan de andere. Niet alleen de uitgebreidheid van de behandeling die u heeft ondergaan speelt een belangrijke rol, maar ook de relatie met uw partner en hoe de seksuele beleving was vóór de behandeling.

Ook door onzichtbare gevolgen van de ziekte of behandeling, zoals vermoeidheid, kan uw behoefte aan seks minder zijn. Daarbij komt nog eens de emotionele verwerking van het (gehad) hebben van kanker. En een vrouw die nog een kind had willen krijgen en bij wie dat door de behandeling niet meer kan, heeft extra tijd nodig om de nieuwe situatie te verwerken. De diagnose kanker brengt voor veel mensen gevoelens van angst en onzekerheid met zich mee. Ook schaamte en machteloosheid kunnen de zin in seks beinvloeden. Soms ook kan er een samenhang zijn tussen seksuele problemen en relatieproblemen.

Partner en naasten

"Kanker heb je niet alleen", is een bekende uitspraak. Vrijwel altijd heeft de kankerbehandeling, en alles wat eraan vooraf is gegaan, ook enorm veel invloed op uw partner en andere naasten. Uw naasten krijgen ook te maken met gevoelens als onzekerheid, angst, machteloosheid, woede en teleurstelling. Het leven ziet er behoorlijk anders uit als uw partner ineens rekening moet gaan houden met het feit dat u niet alles meer aankan, als uw partner dingen voor u moet opvangen en problemen bij u vandaan wil houden. De gevoelens van u en uw partner zullen niet op elk moment gelijk zijn wat soms erg moeilijk kan zijn. Na uw behandeling kan uw partner misschien een terugslag krijgen en steun nodig hebben. Stel u open voor zijn of haar emoties, en trek tijd uit om over elkaars gevoelens te praten. U zult samen op zoek moeten naar een nieuw evenwicht in uw

onderlinge relatie waarbij u zich allebei goed voelt.

Werkhervatting

Als u een baan heeft, moet u uw werkzaamheden misschien tijdelijk aanpassen of stoppen. Overleg hierover met uw specialist en uw bedrijfsarts. Na verloop van tijd kunt u zo mogelijk uw werkzaamheden weer hervatten. Een bepaalde tijd is hiervoor niet te geven, dat is voor iedereen anders.

Erfelijkheid

Baarmoederkanker is, net als de meeste soorten kanker, niet erfelijk. Maar vrouwen met het Lynch syndroom (voorheen HNPCC) - een ziekte die een erfelijke vorm van dikkedarmkanker kan veroorzaken - hebben ook een groter risico op tumoren in andere organen, onder andere in het baarmoederslijmvlies.

Terugkeer van baarmoederkanker

Bij baarmoederkanker is het moeilijk aan te geven wanneer iemand echt genezen is. Ook na een in opzet curatieve behandeling bestaat het risico dat de ziekte terugkomt (recidief). We spreken daarom liever niet van 'genezingspercentages' maar van 'overlevingspercentages'. Daarbij wordt meestal een periode van vijf jaar vanaf de diagnose aangehouden.

Het risico op terugkeer is doorgaans kleiner naarmate de periode dat de ziekte niet aantoonbaar is, langer duurt. De overlevingskans voor een vrouw met baarmoederkanker is afhankelijk van het stadium waarin de ziekte wordt ontdekt.

De overlevingskansen zijn over het algemeen redelijk goed omdat bij 85 procent van de vrouwen de ziekte in een vroeg stadium wordt ontdekt. Dit komt omdat baarmoederkanker vooral voorkomt bij vrouwen die al in de overgang zijn en bloedverlies als klacht in een vrij vroeg stadium optreedt.

- In stadium I is de vijfjaarsoverleving circa 80 tot 95 procent.
- In stadium II is de vijfjaarsoverleving circa 60 tot 80 procent.
- Voor stadium III hangen de vooruitzichten erg af van de specifieke situatie. De behandelend arts kan u hier het beste over informeren. De vijfjaarsoverleving is circa 30 tot 60 procent.
- In stadium IV is de vijfjaarsoverleving circa 10 tot 20 procent.

Overlevingspercentages voor een groep patiënten zijn niet zomaar naar uw individuele situatie te vertalen. Wat u persoonlijk voor de toekomst mag verwachten, kunt u het beste met uw behandelend arts bespreken.

Een recidief is meestal goed te behandelen door middel van een operatie, radiotherapie of chemotherapie.

Kans op een tweede tumor

Er is een aantal risicofactoren dat het ontstaan van baarmoederkanker kan bevorderen, waaronder: overgewicht, en diabetes mellitus (suikerziekte). Uit onderzoek is naar voren gekomen dat bij patiënten juist als gevolg van chemotherapie en/of bestraling kanker kan ontstaan. Het risico op een tweede tumor is echter heel klein. Bij minder dan 1% van de bestraalde patiënten kan een tumor optreden. Dat gebeurt niet op korte termijn, maar meestal pas na 10 tot 30 jaar. Het risico op andere gezondheidsproblemen is veel groter. Daarom is het belangrijk dat u gezond leeft.

Aanbevolen nazorgschema

Wanneer u voor baarmoederkanker bent behandeld, blijft u de eerste twee jaar na de behandeling elke drie maanden onder controle bij de gynaecoloog en/of radiotherapeut. Na twee jaar worden de controles minder en na vijf jaar zijn er geen controles meer nodig.

De controles zijn vooral gericht op het onderzoeken, bespreken en behandelen van eventuele bijwerkingen en gevolgen van de behandeling. Ook wordt onderzocht of de ziekte is teruggekeerd (recidief). De arts verricht inwendig onderzoek en onderzoekt de lymfeklieren in de hals en de liezen. Bij klachten kan verder onderzoek plaatsvinden. U wordt geadviseerd om bij vaginaal bloedverlies contact op te nemen met uw huisarts of specialist.

Nacontroles kunnen spanning en onzekerheid met zich meebrengen, zowel bij u als bij uw naasten. Het kan helpen als u weet wat er bij de verschillende onderzoeken gaat gebeuren.

Lichamelijk onderzoek

Doorgaans zal de gynaecoloog eerst uw buik onderzoeken. Door het voelen en bekloppen van de buik kan vocht in de buik of een eventueel gezwel worden waargenomen.

Daarna doet de gynaecoloog een inwendig onderzoek van de vagina (vaginaal onderzoek) en bevoelt hij de endeldarm (rectaal onderzoek). Dit zijn weliswaar vervelende, maar meestal niet echt pijnlijke onderzoeken.

Bij vaginaal onderzoek brengt de gynaecoloog een of twee vingers in de vagina en legt de andere hand op uw buik. Op deze manier krijgt de arts een indruk van de ligging en de grootte van de organen onder in de buik. De gynaecoloog zal een speculum ('eendenbek') in de vagina inbrengen om de vagina en de vaginatop beter te kunnen zien.

Bij rectaal onderzoek brengt de gynaecoloog een vinger in de endeldarm. Op deze manier probeert hij een indruk te krijgen van de endeldarm, het onderste deel van de buikholte en de organen die zich daar bevinden.

Op indicatie kunnen er aanvullende onderzoeken plaatsvinden:

Echografie van de buikorganen

Bij een echografie van de buikorganen worden de lever, nieren en lymfeklieren in beeld gebracht. De arts let met name op eventuele uitzaaiingen in uw buik.

Echografie is een onderzoek met behulp van geluidsgolven. Deze golven zijn niet hoorbaar, maar de weerkaatsing (echo) ervan maakt organen en/of weefsels zichtbaar op een beeldscherm.

Een eventuele tumor en/of uitzaaiingen kunnen zo in beeld worden gebracht. Tijdens het onderzoek ligt u op een onderzoektafel. Nadat op uw huid een gelei is aangebracht, wordt daarover een klein apparaat bewogen dat geluidsgolven uitzendt. De afbeeldingen op het beeldscherm kunnen op foto's worden vastgelegd.

Echografie is een eenvoudig, niet belastend onderzoek. Soms is het noodzakelijk dat u een volle blaas heeft, waardoor de organen onder in de buik beter in beeld komen.

CT-scan (computertomografie)

Een computertomograaf brengt organen en/of weefsels zeer gedetailleerd in beeld. Bij het maken van een CT-scan wordt gelijktijdig gebruikgemaakt van röntgenstraling en een computer.

Het apparaat heeft een ronde opening waar u, liggend op een beweegbare tafel, doorheen wordt

geschoven. Terwijl de tafel verschuift, maakt het apparaat een serie foto's waarop telkens een ander 'plakje' van het orgaan of weefsel staat afgebeeld. Deze 'doorsneden' geven een beeld van de plaats, grootte en uitbreiding van de (mogelijke) tumor en/of uitzaaiingen. Ook kan de CT-scan informatie opleveren over de toestand van bijvoorbeeld de milt en de lever.

Vaak is een contrastvloeistof nodig. Meestal krijgt u deze vloeistof tijdens het onderzoek in een bloedvat van uw arm gespoten. Contrastvloeistof kan een warm en weeïg gevoel veroorzaken. Sommige mensen worden er een beetje misselijk van. Om ervoor te zorgen dat u hier zo min mogelijk last van heeft, is het advies enkele uren voor het onderzoek niet te eten en te drinken.

MRI (Magnetic Resonance Imaging)

Dit onderzoek maakt gebruik van een magneetveld in combinatie met radiogolven en een computer. De techniek maakt 'dwars- of lengtedoorsneden' van het lichaam zichtbaar, waardoor een eventuele tumor en/of uitzaaiingen in beeld komen.

Tijdens dit onderzoek ligt u in een soort koker. Sommige mensen ervaren het onderzoek daardoor als benauwend.
Een MRI-apparaat maakt nogal wat lawaai. Hiervoor krijgt u oordopjes in. Soms kunt u naar (uw eigen) muziek luisteren. Via de intercom blijft altijd contact bestaan tussen u en de laborant, die tijdens het onderzoek in een andere ruimte is.

Soms wordt tijdens het onderzoek via een bloedvat in uw arm een contrastvloeistof toegediend.

Psychosociale Screening

De diagnose kanker en de daaropvolgende behandeling hebben ingrijpende gevolgen voor u als patiënt en uw naasten. Naast de lichamelijke klachten kunt u vaak emotionele, sociale, en spirituele veranderingen en problemen ervaren. Het is goed om deze veranderingen met uw zorgverlener (bijvoorbeeld uw behandelend arts, verpleegkundige, psycholoog of geestelijk verzorger) te bespreken zodat uw zorgbehoefte in beeld kan worden gebracht. Dit wordt ook wel psychosociale screening genoemd. De zorgverlener kan u, zowel binnen als buiten het ziekenhuis, extra begeleiding bieden.

De manier waarop zorgverleners eventuele psychosociale problemen met u bespreken kan verschillen. Uw zorgverlener kan bijvoorbeeld gebruik maken van de zogenaamde lastmeter. De lastmeter is een vragenlijst waarop u als patiënt de mogelijkheid krijgt om zelf aan uw zorgverlener te laten weten hoe het met u gaat. Het kan u helpen in gesprek te gaan met uw zorgverlener over problemen die u door uw ziekte ervaart.

Het kan zijn dat bepaalde klachten nu nog niet relevant zijn voor u, maar dat deze in de loop van de tijd wel relevant worden. Als u in de loop van de tijd psychosociale problemen wilt bespreken met uw zorgverlener, dan kan dat. U kunt bijvoorbeeld de lastmeter de volgende keer ingevuld meenemen naar uw gesprek met uw zorgverlener. U kunt de lastmeter vinden op de laatste pagina van dit zorgplan.

Bij wie kunt u met uw medische problemen/klachten terecht?

Indien u te maken krijgt met een of meerdere van de gevolgen van baarmoederkanker, kunt u contact opnemen met uw behandelend specialist. Ook wanneer u twijfels heeft kunt u naar uw behandelend specialist en/of huisarts gaan.

Met eventuele psychische en/of seksuele problemen kunt u naast de huisarts ook terecht bij een psycholoog of seksuoloog.

Bij de volgende klachten wordt u geadviseerd contact op te nemen met uw behandelend specialist:

- vaginaal bloedverlies
- bloed bij plassen

Aantekeningen

Datum

Voorlichtingsmaterialen

KWF Kankerbestrijding

Heeft u vragen over kanker van meer algemene aard, of wilt u voor uw bezoek aan uw arts eerst eens met iemand anders over uw vragen praten, dan kunt u onder meer terecht bij het Voorlichtingscentrum van de Nederlandse Kankerbestrijding:
Hulp- en informatielijn 0800 - 022 6622

KWF folders:

- Baarmoederkanker
- Chemotherapie
- Radiotherapie
- Vermoeidheid na kanker
- Als kanker meer is dan je aankunt
- Uitzaaiingen bij kanker
- Kanker en seksualiteit
- Pijnbestrijding bij kanker
- Voeding bij kanker
- Roken en kanker
- Verder leven met kanker

www.kwfkankerbestrijding.nl

Nederlandse Federatie voor Kankerpatiëntenorganisaties (NFK)

Binnen de NFK werken 25 patiëntenorganisaties samen. Zij geven steun en informatie, en komen op voor de belangen van (ex-)kankerpatiënten en hun naasten. De NFK werkt eraan om hun positie in zorg en maatschappij te verbeteren. De NFK en de kankerpatiëntenorganisaties werken samen met en worden gefinancierd door KWF Kankerbestrijding.
Het Platform Vermoeidheid van de NFK zet zich in voor mensen die kanker hebben (gehad) en als gevolg van de ziekte en/of de behandeling kampen met ernstige vermoeidheidsproblemen: www.nfk.nl (klik 'Vermoeidheid' op de homepagina en daarna op 'Lotgenotencontact').

www.nfk.nl

Olijf

Stichting Olijf is een netwerk van en voor vrouwen die gynaecologische kanker hebben (gehad). Dit betekent dat vrouwen met kanker aan baarmoeder(hals), eierstokken, vulva of vagina bij deze patiëntenorganisatie terechtkunnen voor contact met medepatiënten. Over het hele land verspreid zijn vrouwen, allen zelf (ex-)patiënte, bereikbaar voor telefonisch contact. Wie behoefte heeft aan contact of verdere informatie wenst, kan bellen of mailen naar:

Stichting Olijf
Netwerk van vrouwen met gynaecologische kanker
(033) 463 32 99 (ma - do 9.00 - 13.00 uur)
olijf@olijf.nl

Wie nieuwsgierig is naar ervaringen van lotgenoten vindt op www.olijf.nl zowel ervaringsverhalen als een link naar het forum (www.forum.olijf.nl). Voor leden van Stichting Olijf is er een apart forum-gedeelte over baarmoederkanker.

www.olijf.nl

Informatie Centrum Gynaecologie (ICG)

Het ICG heeft als doel om vrouwen de informatie en steun te bieden die ze nodig hebben bij het omgaan met gynaecologische klachten, behandelingen en ingrepen, zodat ze weloverwogen keuzes kunnen maken ten aanzien van producten in de zorg. Dit doen zij op basis van ervaringsdeskundigheid.

www.icgynaecologie.nl

Kanker wie helpt

Op de website van 'Kanker wie helpt' vindt u gedetailleerde informatie over verschillende tumorsoorten en kunt u het zorgaanbod in uw regio bekijken.

www.kankerwiehelpt.nl

Herstel & Balans revalidatieprogramma

Herstel & Balans is een programma voor gezondheidsbevordering en revalidatie voor kankerpatiënten. Het is een combinatie van lichaamstraining en psychosociale begeleiding. Het programma duurt drie maanden en er zijn twee à drie bijeenkomsten per week in een groep van acht tot zestien deelnemers.

Kankerpatiënten die na de behandeling(en) willen werken aan hun herstel en het vinden van een nieuw evenwicht in hun leven, kunnen deelnemen aan het revalidatie programma Herstel & Balans.

Indien u niet in aanmerking komt voor het revalidatieprogramma Herstel & Balans, of het programma past niet bij uw behoeften of indicatie, dan bestaan er andere mogelijkheden voor nazorg. U kunt het beste met uw arts bespreken welke nazorg geschikt voor u is.
www.herstelenbalans.nl

www.herstelenbalans.nl

Nijmeegs Kenniscentrum Chronische Vermoeidheid

Het Nijmeegs kenniscentrum is gespecialiseerd in de behandeling van mensen met chronische vermoeidheid door middel van cognitieve gedragstherapie.
(024) 361 00 30 (ma - do 9.30 - 11.00 uur)

www.umcn.nl/nkcv

Helen Dowling Instituut

Instituut in Utrecht voor psychosociale begeleiding voor alle mensen met kanker, ongeacht de aard en de ernst van de ziekte, de fase van behandeling of de vooruitzichten. Ook voor naasten.

Internettraining van Helen Dowling Instituut:
www.mindermoebijkanker.nl

www.hdi.nl/

Erfocentrum

Het erfocentrum biedt objectieve en betrouwbare informatie over:

- erfelijkheid (DNA, genen, chromosomen)
- erfelijke ziekten en aandoeningen
- zwangerschap, kinderwens en erfelijkheid

www.erfelijkheid.nl

STOET

Stichting opsporing erfelijke tumoren stelt zich ten doel het periodieke onderzoek van familieleden die een verhoogd risico hebben op kanker door een erfelijke belasting landelijk te bevorderen en te coördineren.

www.stoet.nl

De Lastmeter

De last-meter

Invuldatum: .... - .... - .... (dag-maand-jaar)

Hoeveel last heeft u van problemen, klachten, zorgen?

Als eerste
Omcirkel het nummer op onderstaande thermometer dat het best samenvat hoeveel last u de afgelopen week (inclusief vandaag) heeft gehad op lichamelijk, emotioneel, sociaal en praktisch gebied.

10 = extreem veel last

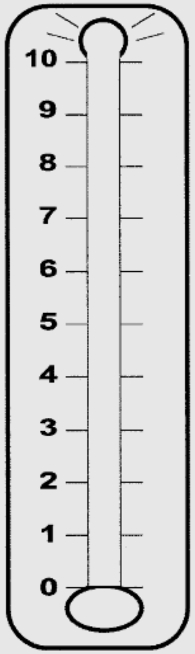

0 = helemaal geen last

Ten tweede
Wilt u voor onderstaande gebieden aangeven of u de afgelopen week (inclusief vandaag) hier moeite mee hebt gehad of problemen bij hebt ervaren.
Wilt u elke vraag beantwoorden?

Ja	Nee	
		**Praktische problemen**
O	O	zorg voor kinderen
O	O	wonen/huisvesting
O	O	huishouden
O	O	vervoer
O	O	werk/school/studie
O	O	financiën
O	O	verzekering
		**Gezins-/sociale problemen**
O	O	omgang met partner
O	O	omgang met kinderen
O	O	omgang met familie/vrienden
		**Emotionele problemen**
O	O	greep hebben op emoties
O	O	herinneren van dingen
O	O	zelfvertrouwen
O	O	angsten
O	O	neerslachtigheid/somberheid
O	O	spanning
O	O	eenzaamheid
O	O	concentratie
O	O	schuldgevoel
O	O	controleverlies
		**Religieuze/spirituele problemen**
O	O	zin van het leven/ levensbeschouwing
O	O	vertrouwen in God/geloof

Ja	Nee	
		**Lichamelijke problemen**
O	O	uiterlijk
O	O	veranderde urine-uitscheiding
O	O	verstopping/obstipatie
O	O	diarree
O	O	eten
O	O	opgezwollen gevoel
O	O	koorts
O	O	mondslijmvlies
O	O	misselijkheid
O	O	droge, verstopte neus
O	O	pijn
O	O	seksualiteit
O	O	droge, jeukerige huid
O	O	slaap
O	O	benauwdheid
O	O	duizeligheid
O	O	praten
O	O	smaakvermogen
O	O	veranderingen in gewicht
O	O	tintelingen in handen/voeten
O	O	wassen/aankleden
O	O	dagelijkse bezigheden
O	O	moeheid
O	O	conditie
O	O	spierkracht

Andere problemen

____

____

Zou u met een deskundige willen praten over uw problemen?

O ja O misschien O nee
